# Supplementary material for: Intronic RNA of yeast RPL22 paralogs acts as an allosteric switch
Source: PLoS One. 2026 Oct 1;21(10):e0359197. doi: 10.1371/journal.pone.0359197 (PMC13630204; doi:10.1371/journal.pone.0359197)
Supplement: Supplementary file 2 [file pone.0359197.s003.pdf]

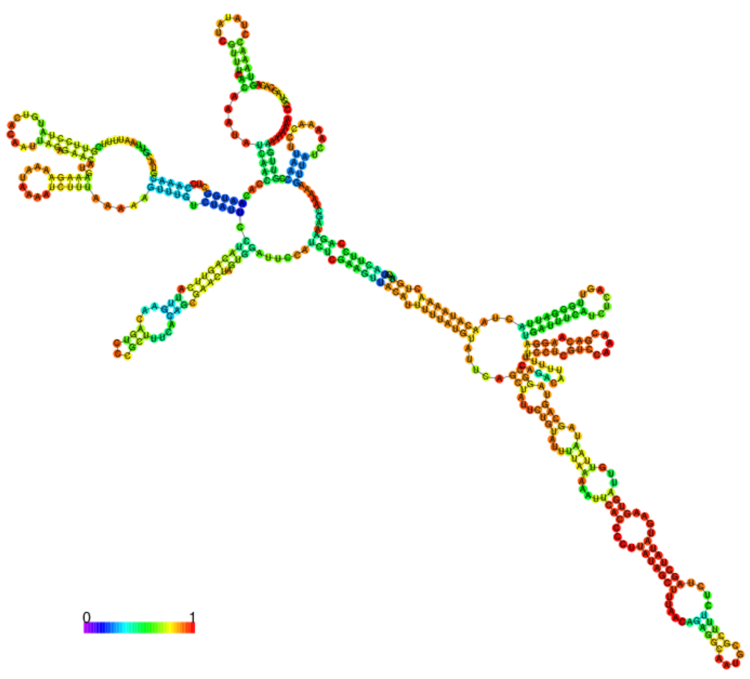

wt

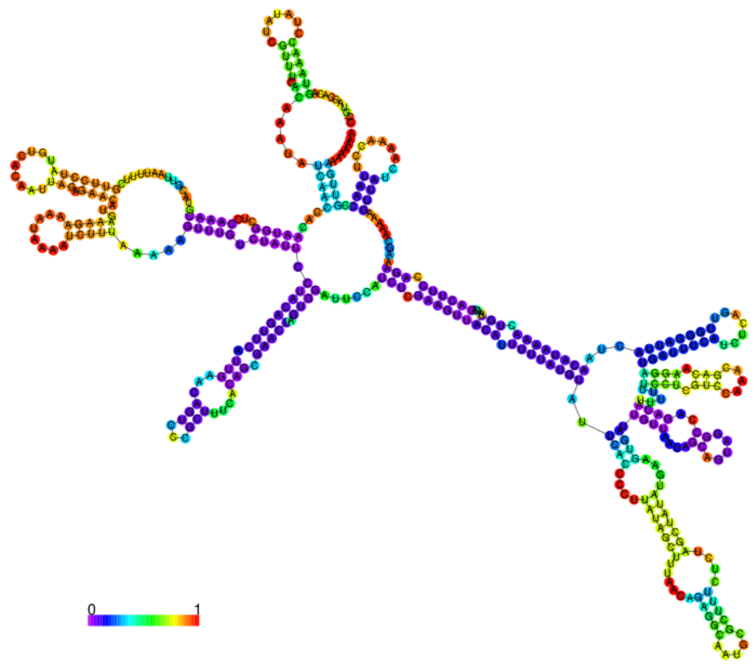

Δ3'C

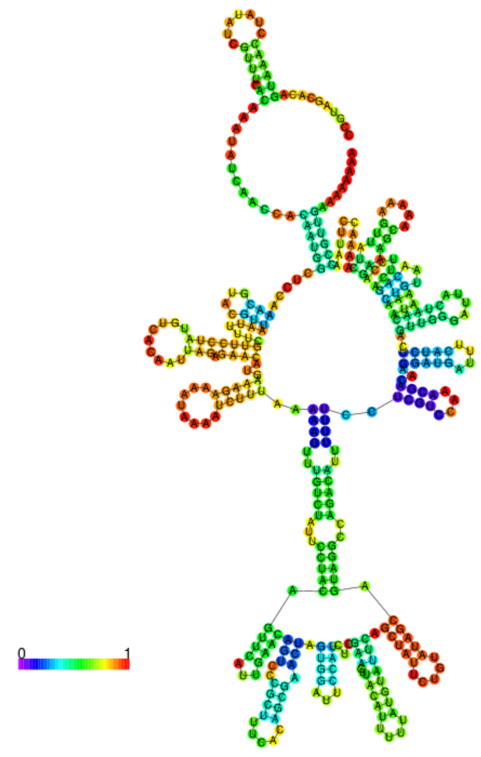

ΔI2

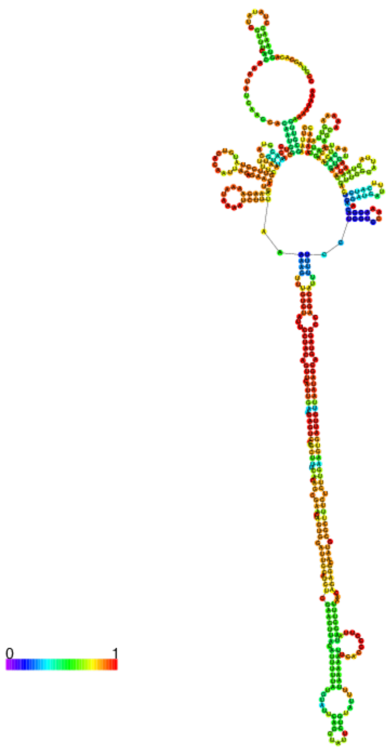

Δ3'clamp

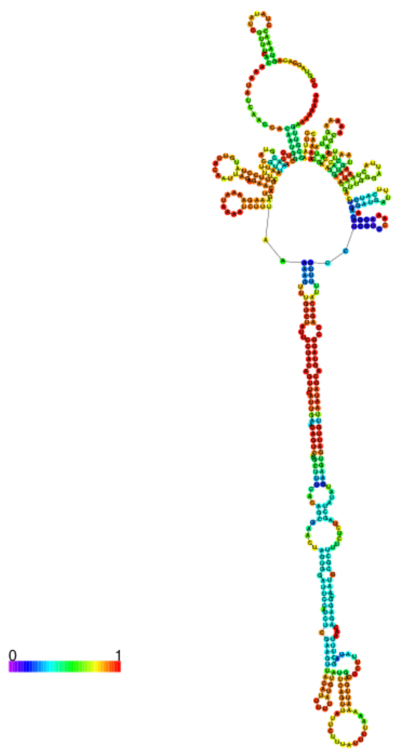

3'CW

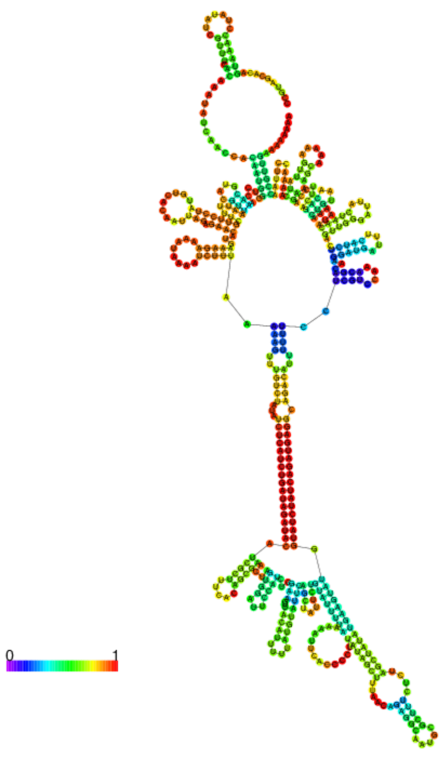

5'CPS

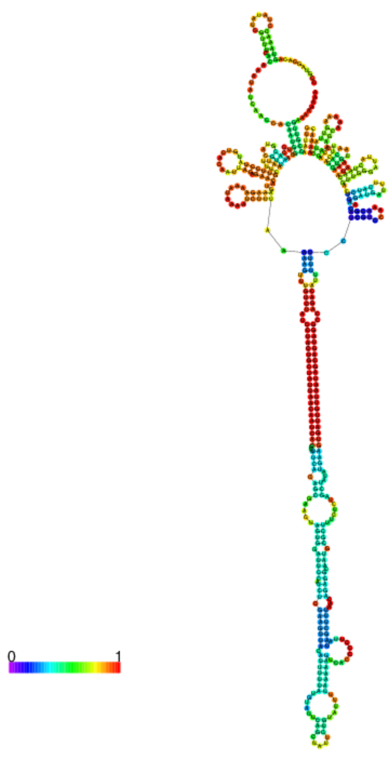

5'CP

S3 Fig RNAfold predicted structures of introns and their mutants tested in Fig. 3. Secondary structures diagrams were generated in RNAfold. The extent of the RNA molecules used for modeling spanned 44 nt in front of the intron and 41 nt after the last nucleotide of the intron. The coloring corresponds to the MFE values indicated under the color bar.
