# Supplementary material for: Intronic RNA of yeast RPL22 paralogs acts as an allosteric switch
Source: PLoS One. 2026 Oct 1;21(10):e0359197. doi: 10.1371/journal.pone.0359197 (PMC13630204; doi:10.1371/journal.pone.0359197)
Supplement: Supplementary file 5 [file pone.0359197.s006.pdf]

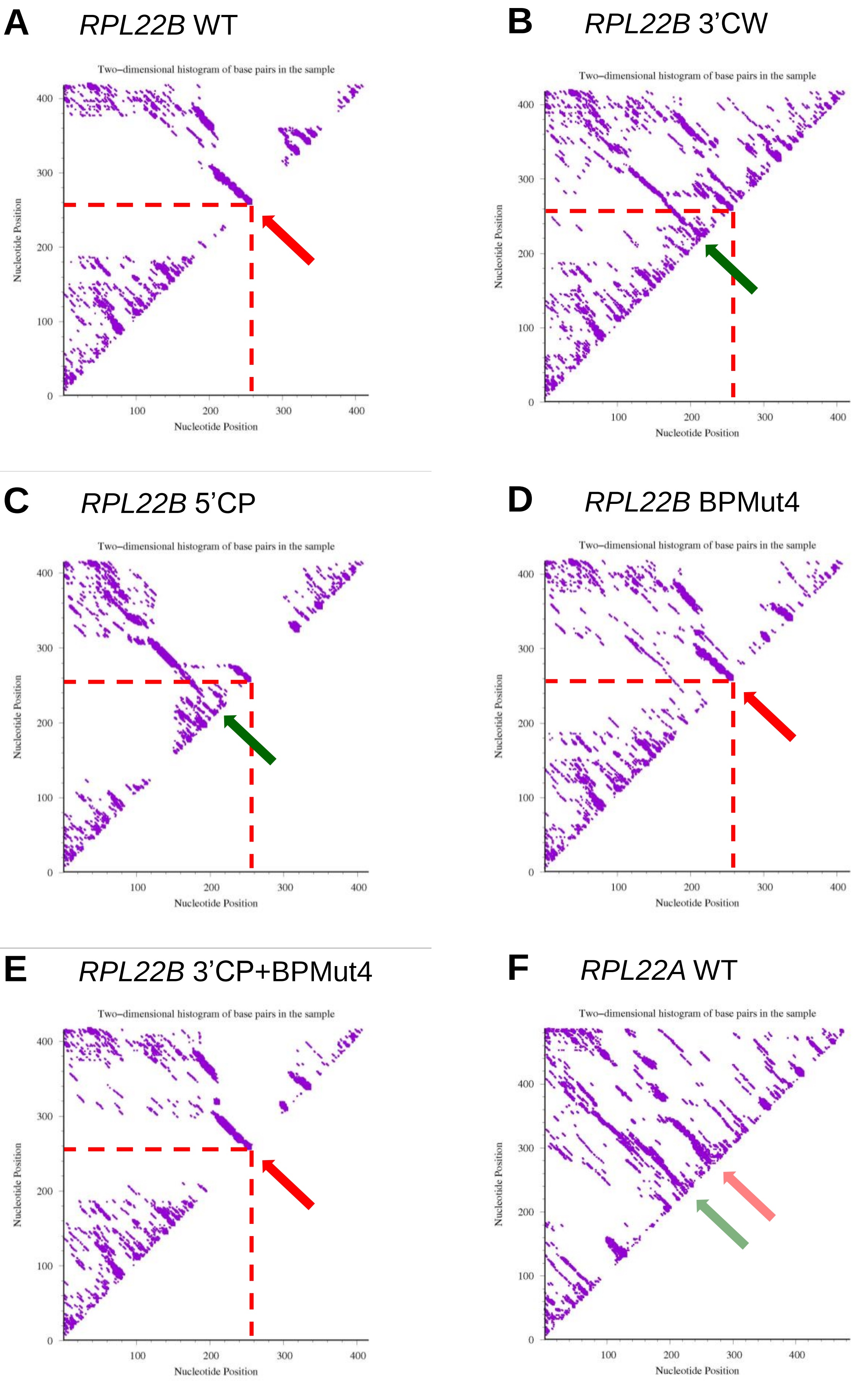

A
RPL22B WT
B
RPL22B 3'CW
C
RPL22B 5'CP
D
RPL22B BPMut4
E
RPL22B 3'CP+BPMut4
F
RPL22A WT
Two–dimensional histogram of base pairs in the sample
Nucleotide Position
Nucleotide Position

**S6 Fig Base pairing probabilities of Sfold predicted RNA ensembles of *RPL22* introns.** Two-dimensional diagrams of base pairing probabilities from a statistical sample of structures were generated by Sfold (*S*rna; [4]) for *RPL22B* WT **(A)**, 3'CW **(B)**, 5'CP **(C)**, BPMut4 **(D)**, 3'CP+BPMut4, and *RPL22A* WT **(D)**. The extent of the sequence used for modeling was the same as in Figs. S2-S5. The position of the apical end of the clamp in *RPL22B* is marked by dashed lines. The densities corresponding to the 3'COMP/CONS (I) and 5'COMP/CONS (P) base pairing are indicated by the red and green arrows, respectively.

[4] Ding Y. A statistical sampling algorithm for RNA secondary structure prediction. Nucleic Acids Research 2003;31:7280–301. https://doi.org/10.1093/nar/gkg938.
