# Supplementary material for: Intronic RNA of yeast RPL22 paralogs acts as an allosteric switch
Source: PLoS One. 2026 Oct 1;21(10):e0359197. doi: 10.1371/journal.pone.0359197 (PMC13630204; doi:10.1371/journal.pone.0359197)
Supplement: Supplementary file 6 [file pone.0359197.s007.pdf]

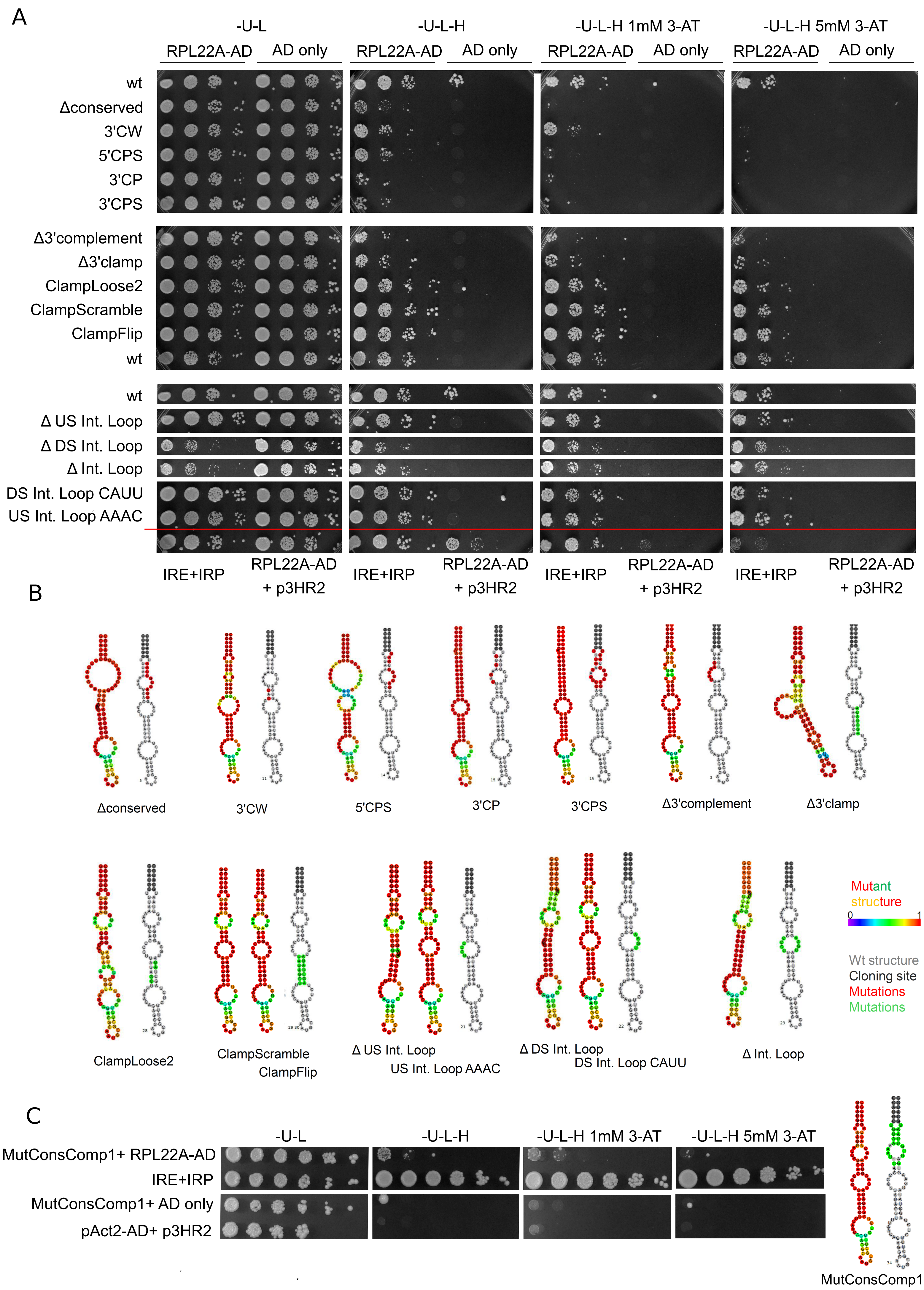
A
-U-L
-U-L-H
-U-L-H 1mM 3-AT
-U-L-H 5mM 3-AT
RPL22A-AD
AD only
wt
Δconserved
3'CW
5'CPS
3'CP
3'CPS
Δ3'complement
Δ3'clamp
ClampLoose2
ClampScramble
ClampFlip
wt
wt
Δ US Int. Loop
Δ DS Int. Loop
Δ Int. Loop
DS Int. Loop CAUU
US Int. Loop AAAC
IRE+IRP
RPL22A-AD + p3HR2
B
Δconserved
3'CW
5'CPS
3'CP
3'CPS
Δ3'complement
Δ3'clamp
ClampLoose2
ClampScramble
ClampFlip
Δ US Int. Loop
US Int. Loop AAAC
Δ DS Int. Loop
DS Int. Loop CAUU
Δ Int. Loop
Mutant structure
0
1
Wt structure
Cloning site
Mutations
Mutations
C
-U-L
-U-L-H
-U-L-H 1mM 3-AT
-U-L-H 5mM 3-AT
MutConsComp1+ RPL22A-AD
IRE+IRP
MutConsComp1+ AD only
pAct2-AD+ p3HR2
MutConsComp1

S7 Fig Yeast three-hybrid analyses of Rpl22A binding to the I2 fragment of the intronic *RPL22B* RNA.

(A) The intronic region of *RPL22B* between nt 165 and 236 was tested for its ability to interact with the Rpl22 protein in a yeast three-hybrid assay. Ten-fold serial dilutions of cells were spotted on plates with increasing concentrations of the metabolic retarder 3-aminotriazole (3-AT). '-U', '-L', and '-H' denote the absence of uracil, leucine, and histidine in the medium. Cells with activation domain expressed alone (AD only; right parts of the panels) did not grow in the absence of histidine. Positive (IRE+IRP; [5]) and negative controls (Rpl22-AD with empty plasmid p3HR2) are placed in the last row below the red line. The RNA internal loop mutants supported cell growth to the same extent as the construct containing the WT intron at 1 to 5 mM 3-aminotriazole.

(B) Predictions of stem loop formation in the I2 region of the *RPL22B* intron (RNAfold; [1]). The extent of the RNA molecules used for modeling spanned 44 nt in front of the intron and 41 nt after the last nucleotide of the intron. The fragment spanning nucleotides 165 and 236 is shown. For each mutant, two structures are shown: (i) the predicted structure (left; nucleotide color reflects the probability of the particular arrangement) and (ii) the WT template (in grayscale) with manipulated nucleotides highlighted in red (for splicing inhibitory mutants) and green (for splicing permissive manipulations).

(C) Yeast three hybrid analysis of MutConsComp1 was done as in (A). Prediction of stem loop formation is shown to the right.

[1] Lorenz R, Bernhart SH, Zu Siederdissen CH, Tafer H, Flamm C, Stadler PF, et al. ViennaRNA Package 2.0. Algorithms for Molecular Biology 2011;6:26. https://doi: 10.1186/1748-7188-6-26.

[5] SenGupta DJ, Zhang B, Kraemer B, Pochart P, Fields S, Wickens M. A three-hybrid system to detect RNA-protein interactions in vivo. Proceedings of the National Academy of Sciences 1996;93:8496–501. https://doi: 10.1073/pnas.93.16.8496.
