# Supplementary material for: Intronic RNA of yeast RPL22 paralogs acts as an allosteric switch
Source: PLoS One. 2026 Oct 1;21(10):e0359197. doi: 10.1371/journal.pone.0359197 (PMC13630204; doi:10.1371/journal.pone.0359197)
Supplement: Supplementary file 7 [file pone.0359197.s008.pdf]

```
Rpl22A    MAPN------TSRKQKIAKTFTVDVSSPTENGVFDPASYAKYLIDHIKVEGAVGNLGNAVTVTEDGTV   62
Rpl22B    MAPN------TSRKQKVIKTLTVDVSSPTENGVFDPASYSKYLIDHIKVDGAVGNLGNAIEVTEDGSI   62
KlRpl22   MAPN------TARKQKITKTFTVDVSSPTENGVFDPASYAKYLIDHIKVEGHVGNLGQAITVEEDGSV   62
CgRpl22   MAPN------TARKQKVVKTFTVDVSAPTENGVFDPASYAKYLIDHIKVENVVGNLGNAITVEEDGSV   62
CaRpl22   MAPV------TSKKSKSVKKFVVDVAAPVENDVFDQESYVKYLVEHVKVDGIVGNLGNDISITAESDN   62
SpRpl22   ---------MVKKNTKVSNKYIIDATAAVNDKIFDVAAFEKYLIDRIKVDGKTGNLGSSVSITEEGNK   59
DhRpl22   MAPI------TTKKNTAAKKLVVDTSAPTENGVFDQESYVKFLIENIKVEGIPGNLGNSIVTIERSKS   62
HsRpl22   MAPVKKLVVKGGKKKKQVLKFTLDCTHPVEDGIMDAANFEQFLQERIKVNGKAGNLGGGVVVSREGSS   68
YlRpl22   MAPV------KTQ---KANKYTVDCKAPSADGIFDVSSFEKFLTERIKVEGRTNQLGEDIKVSSNGDI   59
                      :      .  :*      : ::*   : ::* :.:**:.  .:**  :     .

Rpl22A    V---TVVSTAKFSGKYLKYLTKKYLKKNQLRDWIRFVSTKTNEYRLAFYQVTPEEDEEEDEE-  121
Rpl22B    V---TVVSSAKFSGKYLKYLTKKYLKKNQLRDWIRFVSIRQNQYKLVFYQVTPEDADEEEDDE  122
KlRpl22   V---TIVSTTKFSGKYLKYLTKKYLKKNQLRDWIRFVSTKTNEYKLAFYQITPEDEEEEEDEE  122
CgRpl22   V---TIVSTTKFSGKYLKYLTKKYLKKNQLRDWIRFVSTKTNSYRLAFYQVTPEEEEEDEE--  120
CaRpl22   KVVVVVSGNGSFSGKYLKYLTKKYLKKNQIRDWIRFVSVKQNQYKLQFYAVAEDDEEEEDEE-  124
SpRpl22   K--IAVIAHIDFSGRYLKYLTKKFLKKHSLRDWLRVVSTKKGVYELRYYNVVVGNDEEEQ---  117
DhRpl22   V---VVVSNTKFSGKYLKYLTKRYLKKNQIRDWIRFVSVKQNQYQLQFYSVADDEEEEDEE--  120
HsRpl22   K--ITVTSEVPFSKRYLKYLTKKYLKKNNLRDWLRVVANSKESYELRYFQINQDEEEEEDED-  128
YlRpl22   V---TVVSTTQFSGKYLKYLTKKYLKKQQLRDWIRVISTSKGNYTLKFYNVVANEEDEE----  115

              .: .   ** :*******::***:.:***:*.::     * * :: :   : :*:
```

S8 Fig Sequence alignment of *RPL22* homologs tested in this study. Rpl22 sequences are shown from top to bottom: *Saccharomyces cerevisiae* Rpl22A (ScRpl22A), *Saccharomyces cerevisiae* Rpl22B (ScRpl22B), *Kluyveromyces lactis* Rpl22 (KlRpl22), *Candida glabrata* Rpl22 (CgRpl22), *Candida albicans* Rpl22 (CaRpl22), *Schizosaccharomyces pombe* Rpl22 (SpRpl22), *Debaryomyces hansenii* Rpl22 (DhRpl22), *Homo sapiens* Rpl22 (HsRpl22) and *Yarrowia lipolytica* Rpl22 (YlRpl22). The segment corresponding to the RNA binding helix is highlighted in yellow. Lysines that were mutated to glutamates in $Rpl22A_{mut}$ and $Rpl22B_{mut}$ are highlighted in red. Asterisks (*) indicate identical amino acids at a given position; colons (:) and periods (.) indicate strong and weak amino acid conservation, respectively. The alignment was generated using Clustal Omega MSA (https://www.ebi.ac.uk/Tools/msa/clustalo/).
