# Supplementary material for: Intronic RNA of yeast RPL22 paralogs acts as an allosteric switch
Source: PLoS One. 2026 Oct 1;21(10):e0359197. doi: 10.1371/journal.pone.0359197 (PMC13630204; doi:10.1371/journal.pone.0359197)
Supplement: Supplementary file 8 [file pone.0359197.s009.pdf]

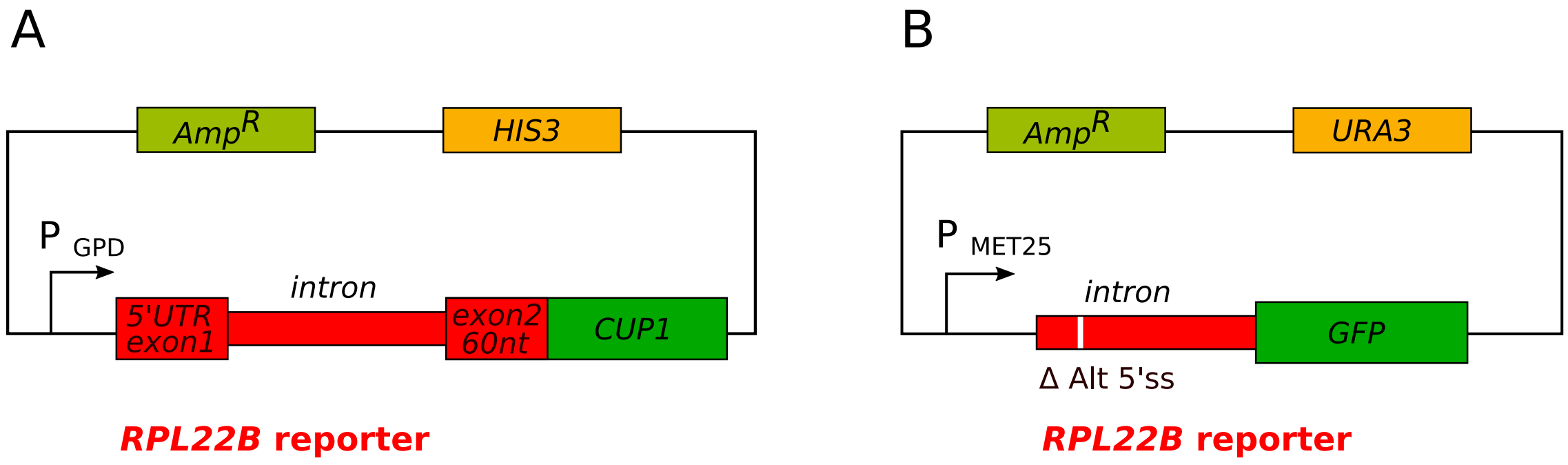
A
AmpR
HIS3
P GPD
intron
5'UTR exon1
exon2 60nt
CUP1
RPL22B reporter
B
AmpR
URA3
P MET25
intron
GFP
Δ Alt 5'ss
RPL22B reporter

S9 Fig The reporter constructs used to analyze RPL22B splicing *in vivo*.

(A) In this study, we used reporter splicing constructs based on the p423GPD backbone with *RPL22B* intron surrounded by its own 5'UTR, exon 1 and 60 nts of exon 2, which were fused in frame with the *CUP1* reporter. Alternative 5'ss was preserved in all constructs unless indicated. **(B)** In the study of Gabunilas and Chanfreau [6], the reporter plasmid was derived from pUG23. *The RPL22B* intron was inserted in front of the *GFP* reporter without the surrounding sequences. In most of the constructs analyzed, the alternative 5'ss was deleted.

[6] Gabunilas J, Chanfreau G. Splicing-Mediated Autoregulation Modulates Rpl22p Expression in Saccharomyces cerevisiae. PLoS Genet 2016;12:e1005999. https://doi: 10.1371/journal.pgen.1005999.
