# Supplementary material for: Intronic RNA of yeast RPL22 paralogs acts as an allosteric switch
Source: PLoS One. 2026 Oct 1;21(10):e0359197. doi: 10.1371/journal.pone.0359197 (PMC13630204; doi:10.1371/journal.pone.0359197)
Supplement: Supplementary file 9 [file pone.0359197.s010.pdf]

A

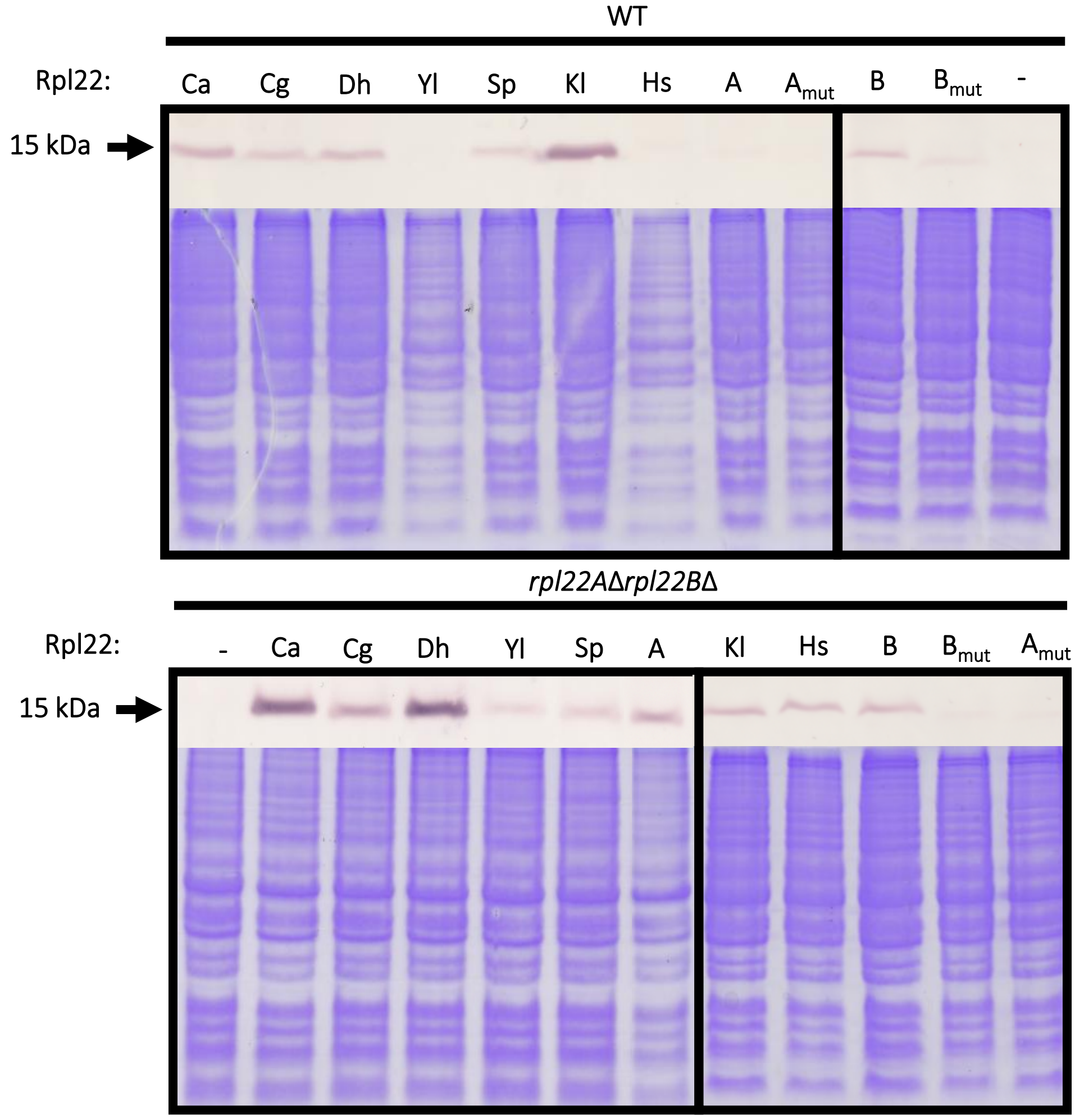

WT
Rpl22:
Ca
Cg
Dh
Yl
Sp
Kl
Hs
A
A mut
B
B mut
-
15 kDa
rpl22AΔrpl22BΔ
Rpl22:
-
Ca
Cg
Dh
Yl
Sp
A
Kl
Hs
B
B mut
A mut
15 kDa

B

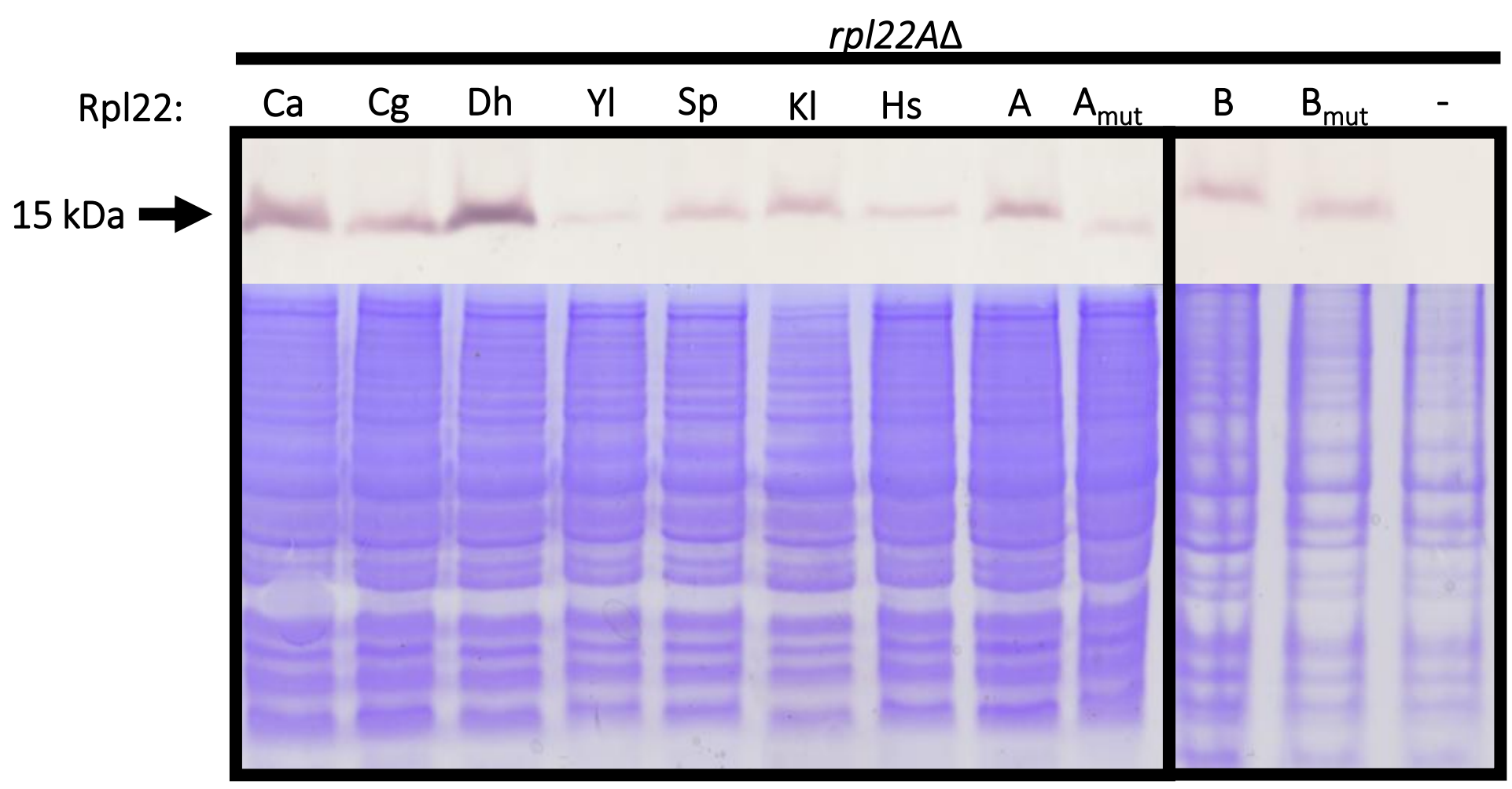

rpl22AΔ
Rpl22:
Ca
Cg
Dh
Yl
Sp
Kl
Hs
A
A mut
B
B mut
-
15 kDa

C

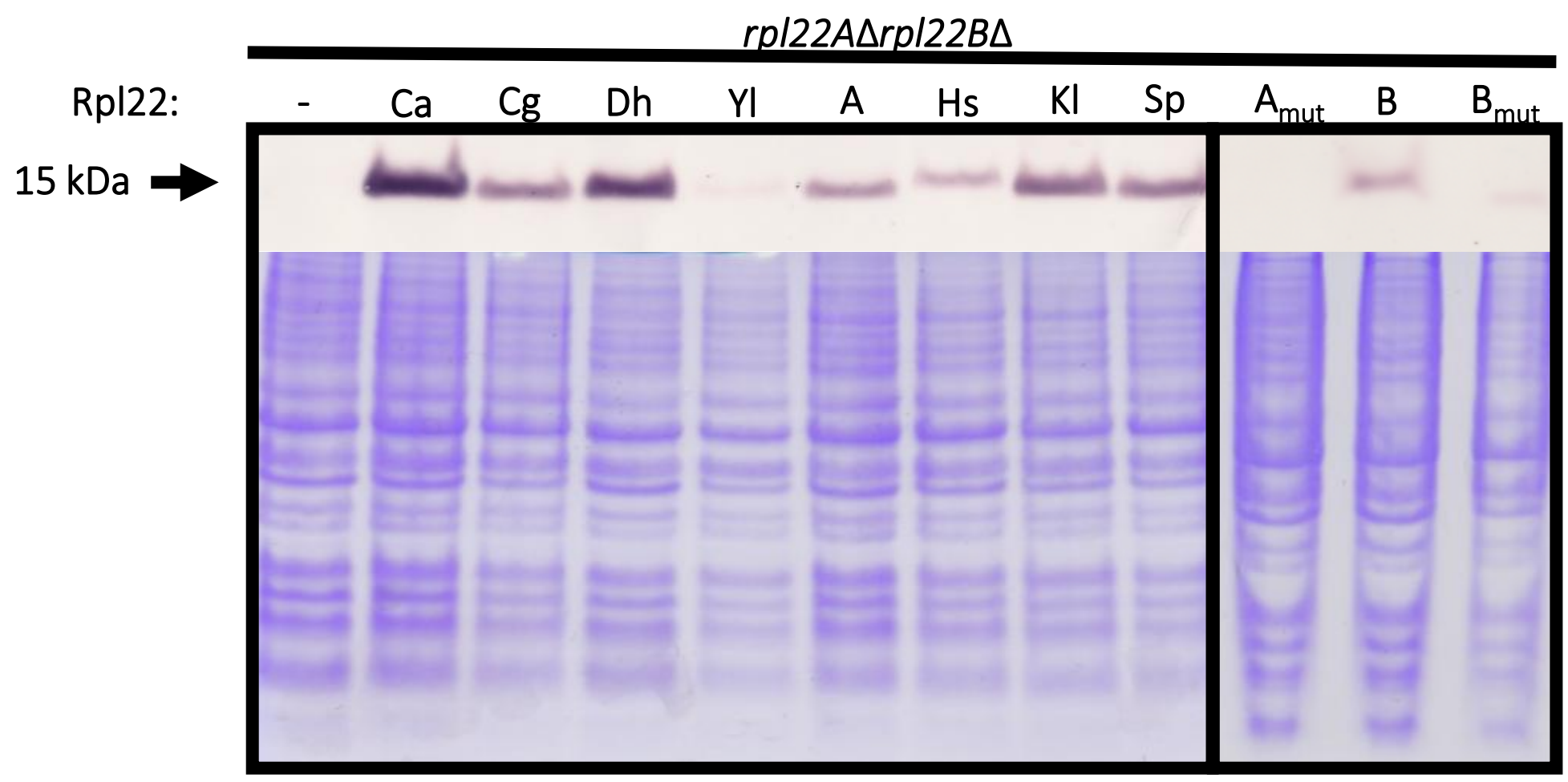

rpl22AΔrpl22BΔ
Rpl22:
-
Ca
Cg
Dh
Yl
A
Hs
Kl
Sp
A mut
B
B mut
15 kDa

S10 Fig Levels of heterologous Rpl22 proteins in the experiments of Fig. 7.

Protein extracts were prepared from exponentially growing cells of the analyzed strains, separated on 10% tris-tricine gels, and transferred to cellulose membranes. Panels show the results of Western blot detection along with the corresponding tris-tricine gels after transfer. Immunodetection of 6xHis-tagged Rpl22 was performed using a primary antibody against the 6xHis tag (6xHis Tag Monoclonal Antibody HIS.H8; Thermo Scientific) and a secondary antibody conjugated to alkaline phosphatase (Goat Anti-Mouse IgG (H+L) AP Conjugate; BIORAD).

(A) Growth rate complementation experiment in Fig. 7B. Different *RPL22* genes were expressed as intronless versions under the control of the *ADH1* promoter (plasmid pVTU260) in *rpl22a*Δ *rpl22b*Δ and WT *S. cerevisiae cells* and evaluated. The Rpl22 proteins tested included Rpl22A (A), the binding-dead mutant of Rpl22 (A_{mut}) and the homologs from *Candida albicans* (Ca), *Candida glabrata* (Cg), *Debaryomyces hanseni* (Dh), *Yarrowia lipolytica* (Yl), *Schizosaccharomyces pombe* (Sp), *Kluyveromyces lactis* (Kl) and *Homo sapiens* (Hs). The expected molecular weight of the detected protein was approximately 15 kDa (indicated by arrows). Protein extracts from cells carrying the empty pVTU260 vector were used as a negative control (–).

(B) Splicing analysis experiment in Fig. 7C. The *RPL22* homologs were expressed in the *rpl22a*Δ strain as in (A).

(C) qPCR measurement of *RPL22B* mRNA in Fig. 7D. The *RPL22* homologs were expressed in the *rpl22a*Δ *rpl22b*Δ background as in (A). Negative controls included extracts from cells containing the empty pVTU260 vector and the reporter plasmid p423GPD_*RPL22B-CUP1*.
