# Supplementary material for: Intronic RNA of yeast RPL22 paralogs acts as an allosteric switch
Source: PLoS One. 2026 Oct 1;21(10):e0359197. doi: 10.1371/journal.pone.0359197 (PMC13630204; doi:10.1371/journal.pone.0359197)

S11 Fig   Raw images of gels and blots.

Originals to Fig 2A - control SPT15 detection

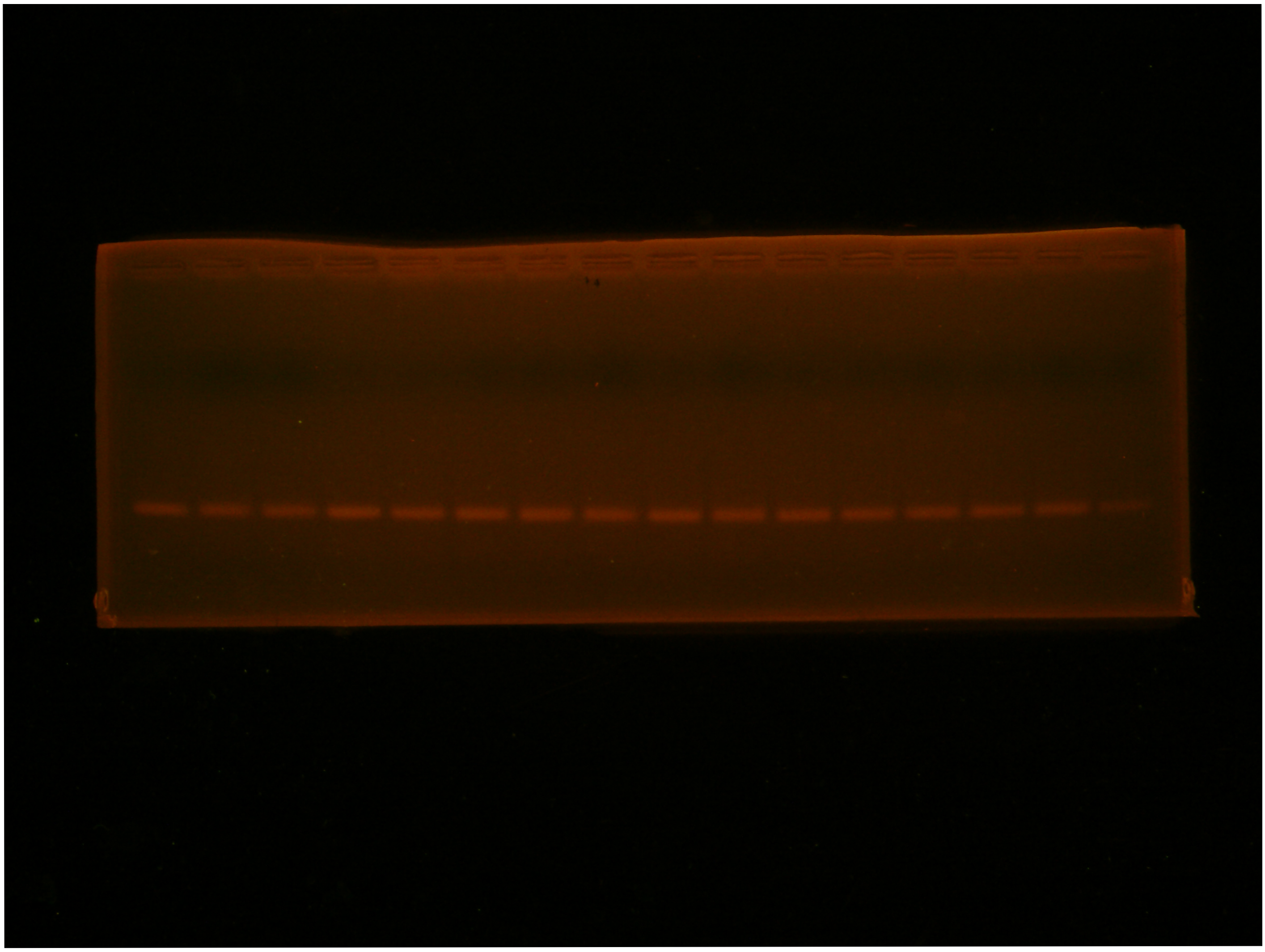

RPL22B intron	Δ5'C		ΔCons		WT		WT		5'CW		3'CP		3'CPS		WT	
Rpl22	+	-	+	-	+	-	+	-	+	-	+	-	+	-	+	-

Originals to Fig 2A and Fig 5A - RPL22B detection

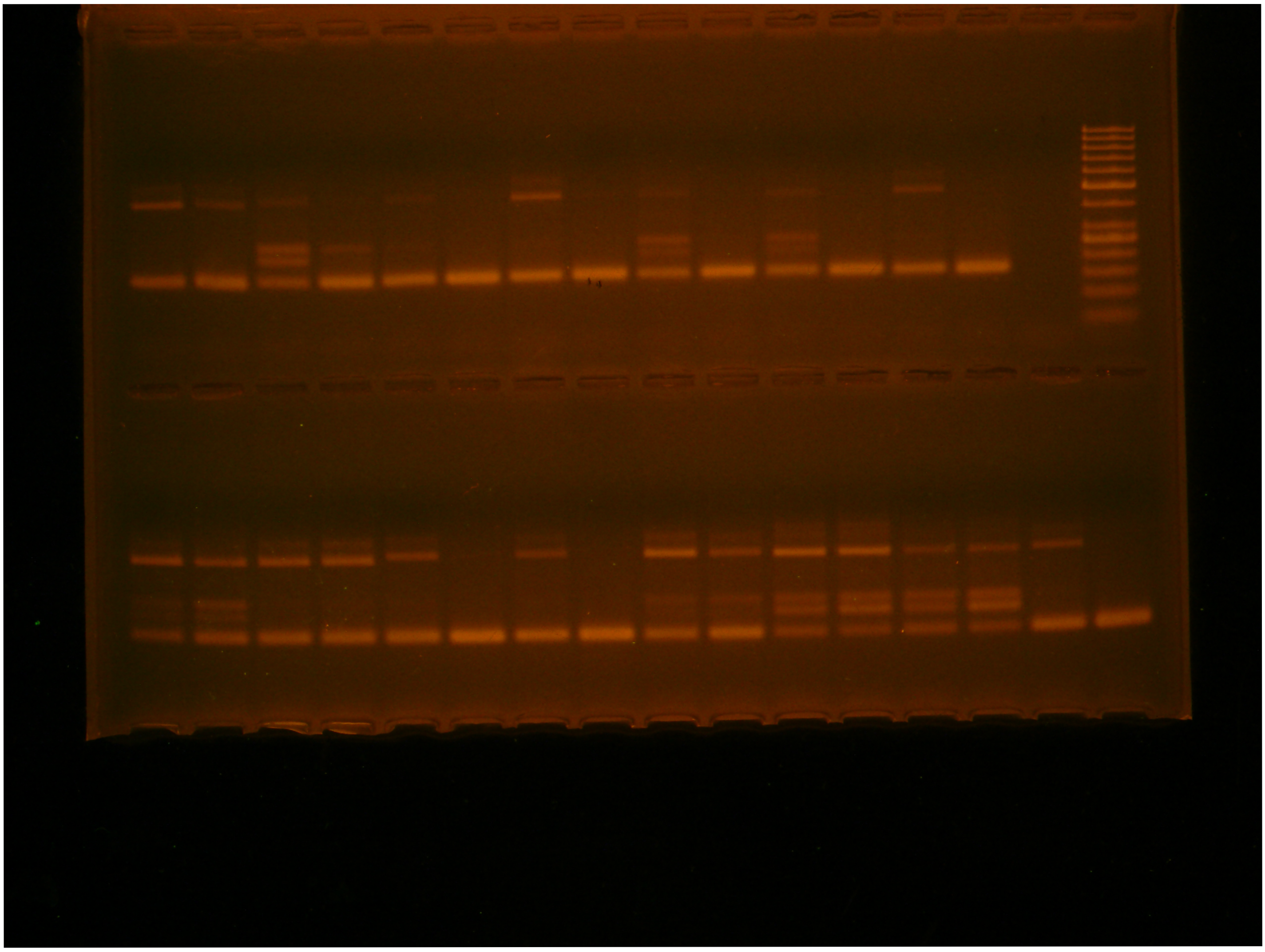

RPL22B intron	Δ Alt 5'ss	Alt 5'ss cons	5'ss cons	WT	Both 5'ss cons	5'CP Alt 5'ss cons	WT	neg ctrl	M
Rpl22	+ -	+ -	+ -	+ -	+ -	+ -	+ -		

RPL22B intron	Δ5'C	ΔCons	WT	WT	5'CW	3'CP	3'CPS	WT
Rpl22	+ -	+ -	+ -	+ -	+ -	+ -	+ -	+ -

Originals to Fig 2C - RPL22A and SPT15 detection

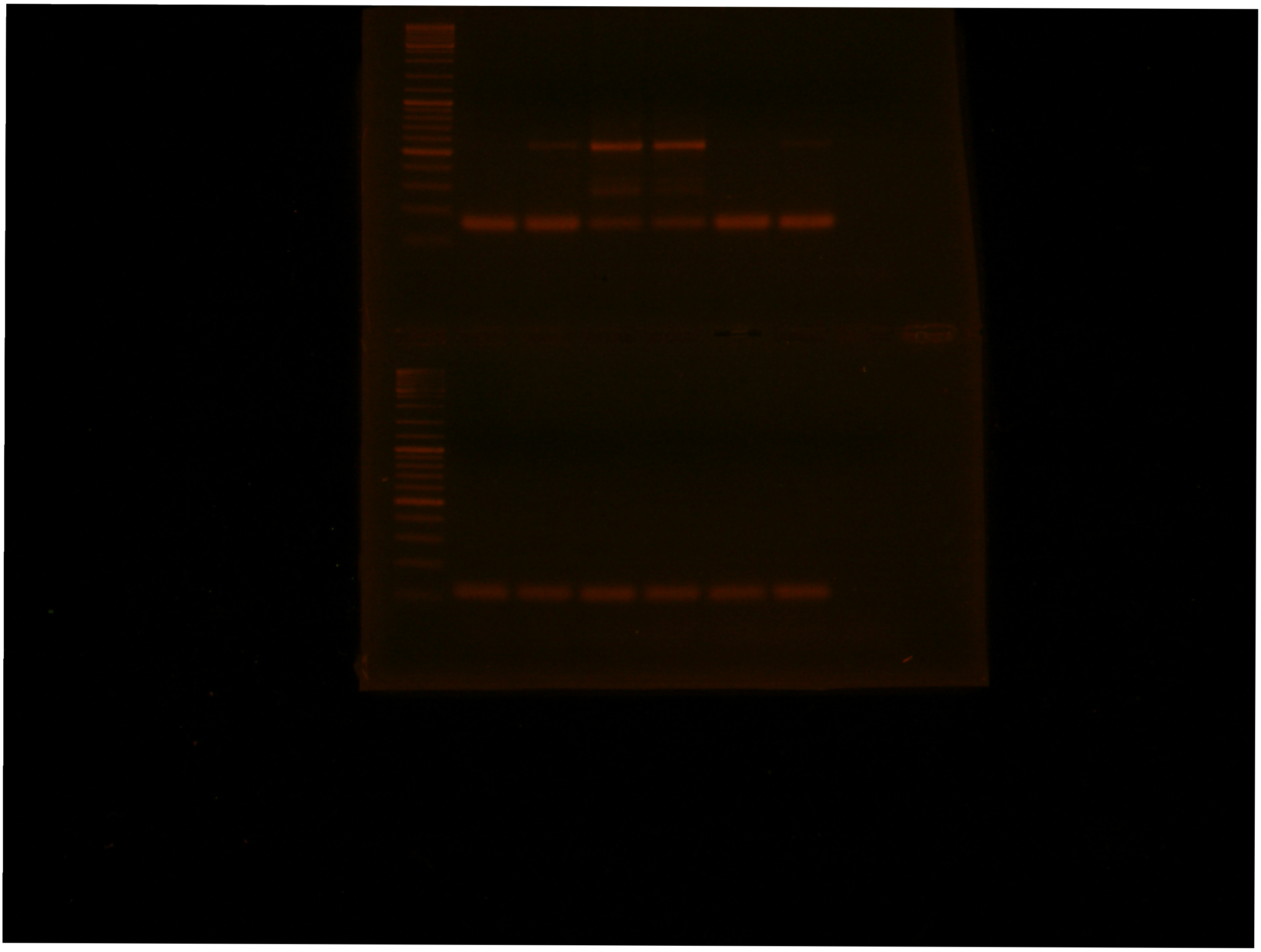

RPL22A intron	M	WT		3'CP		5'CP		Neg. ctrl
Rpl22		-	+	-	+	-	+	

RPL22A intron	M	WT		3'CP		5'CP		Neg. ctrl
Rpl22		-	+	-	+	-	+	

Originals to Fig 2D - RPL22B and SPT15 detection

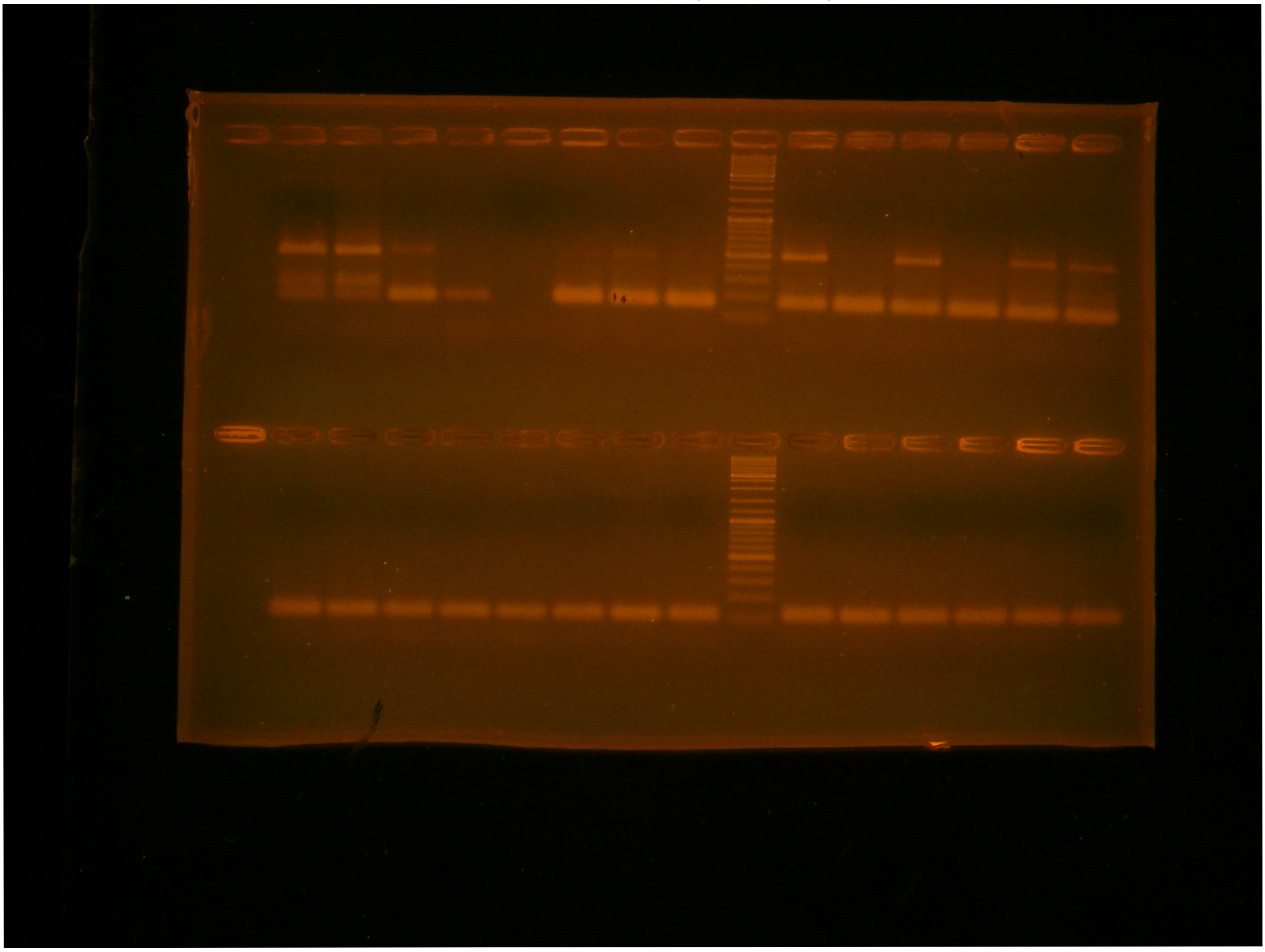

	1	2	3	4	5	6	7	8	9	10	11	12	13	14	15	16
RPL22B intron	Empty	MutCons Comp1		4-9 unrelated samples						M	WT		13-16 unrelated samples			
Rpl22		+	-								+	-				

Upper part - RPL22B
Lower part - SPT15

Originals to Fig 3A and 5Cpart1- control SPT15 detection

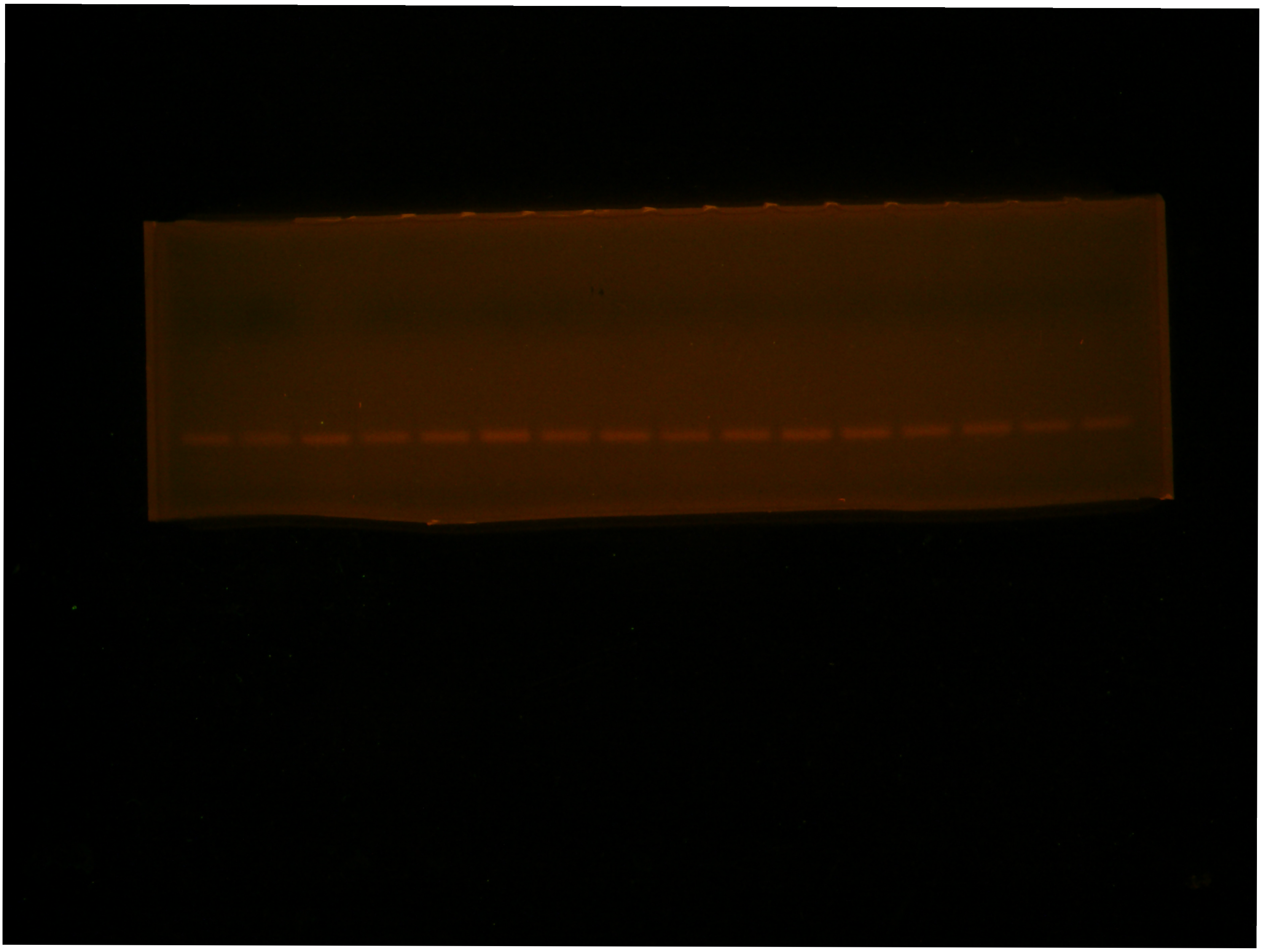

RPL22B intron	Δ3'C		ΔI2		Δ3'Clamp		WT		3'CW		5'CPS		5'CP		WT	
Rpl22	+	-	+	-	+	-	+	-	+	-	+	-	+	-	+	-

Originals to Fig 3A and 5Cpart1- RPL22B detection

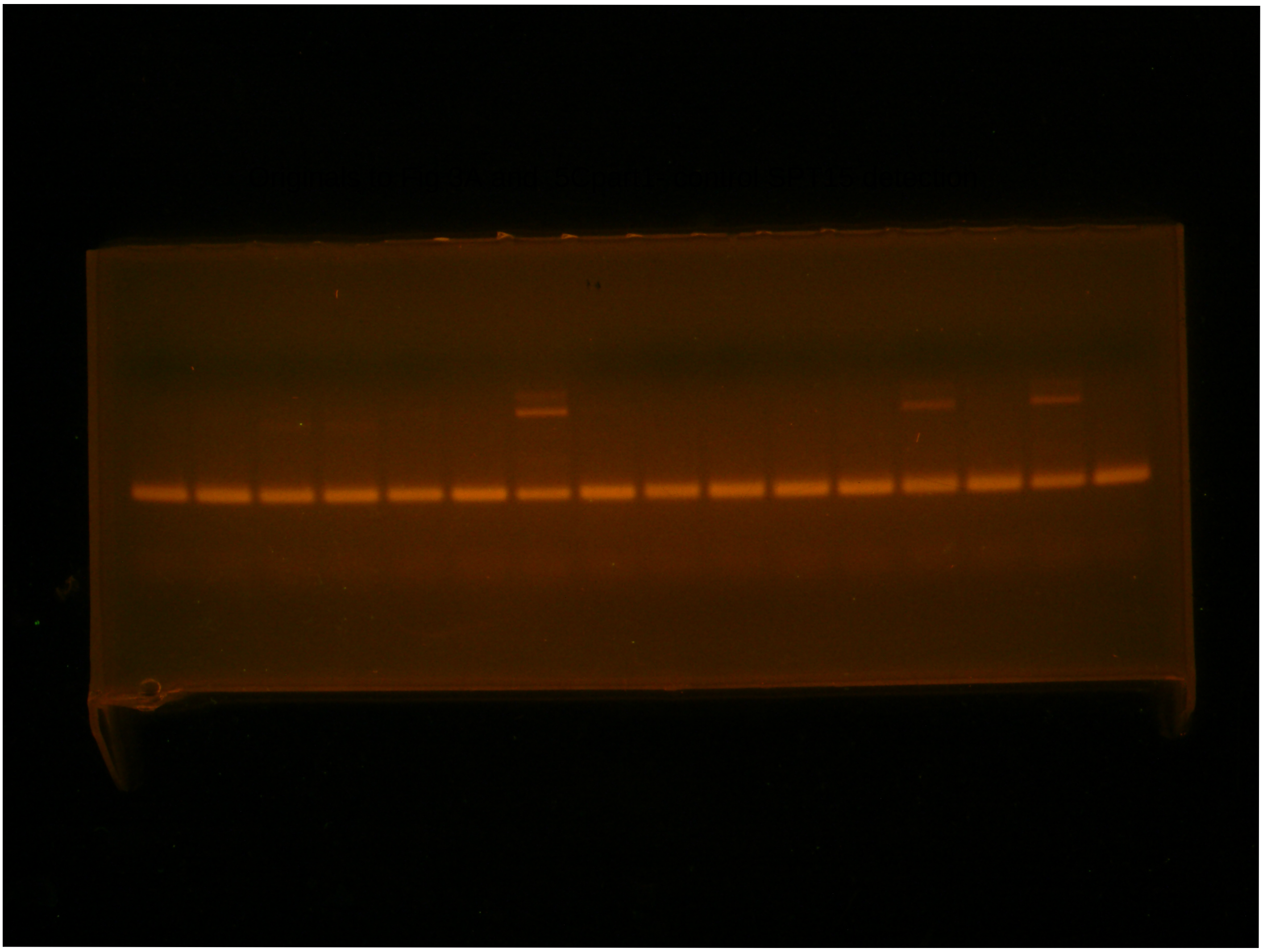

RPL22B intron	Δ3'C		ΔI2		Δ3'Clamp		WT		3'CW		5'CPS		5'CP		WT	
Rpl22	+	-	+	-	+	-	+	-	+	-	+	-	+	-	+	-

Originals to Fig 4A part 1- control SPT15 detection

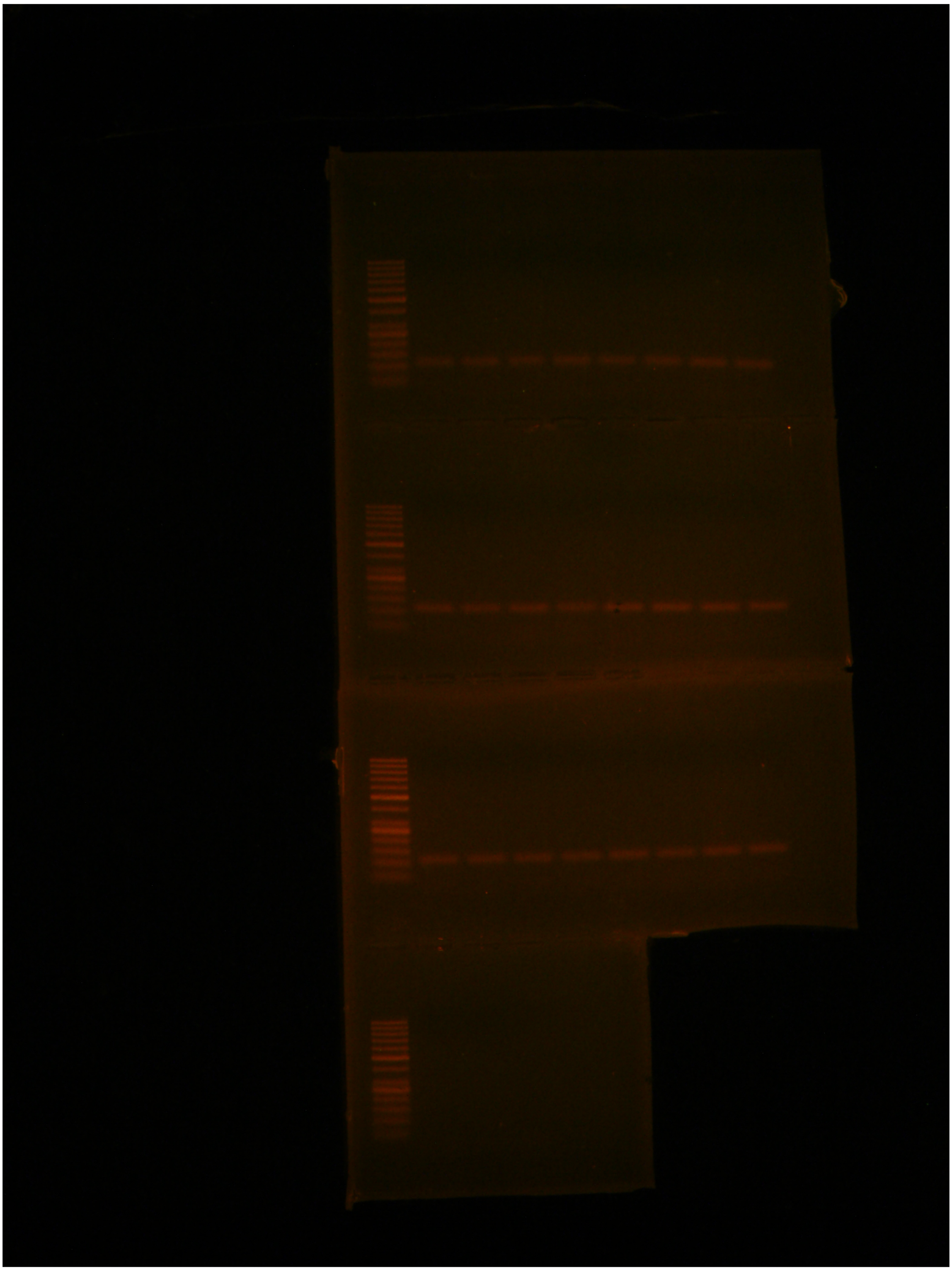

1 2 3 4 5 6 7 8 9 10

1 - 10 Unrelated samples

1 - 10 Unrelated samples

RPL22B intron<br>Rpl22	M	Mutclamp2<br>+ -	4 - 10 Unrelated samples

1 - 10 Unrelated samples

Originals to Fig 4A part 1- RPL22B detection

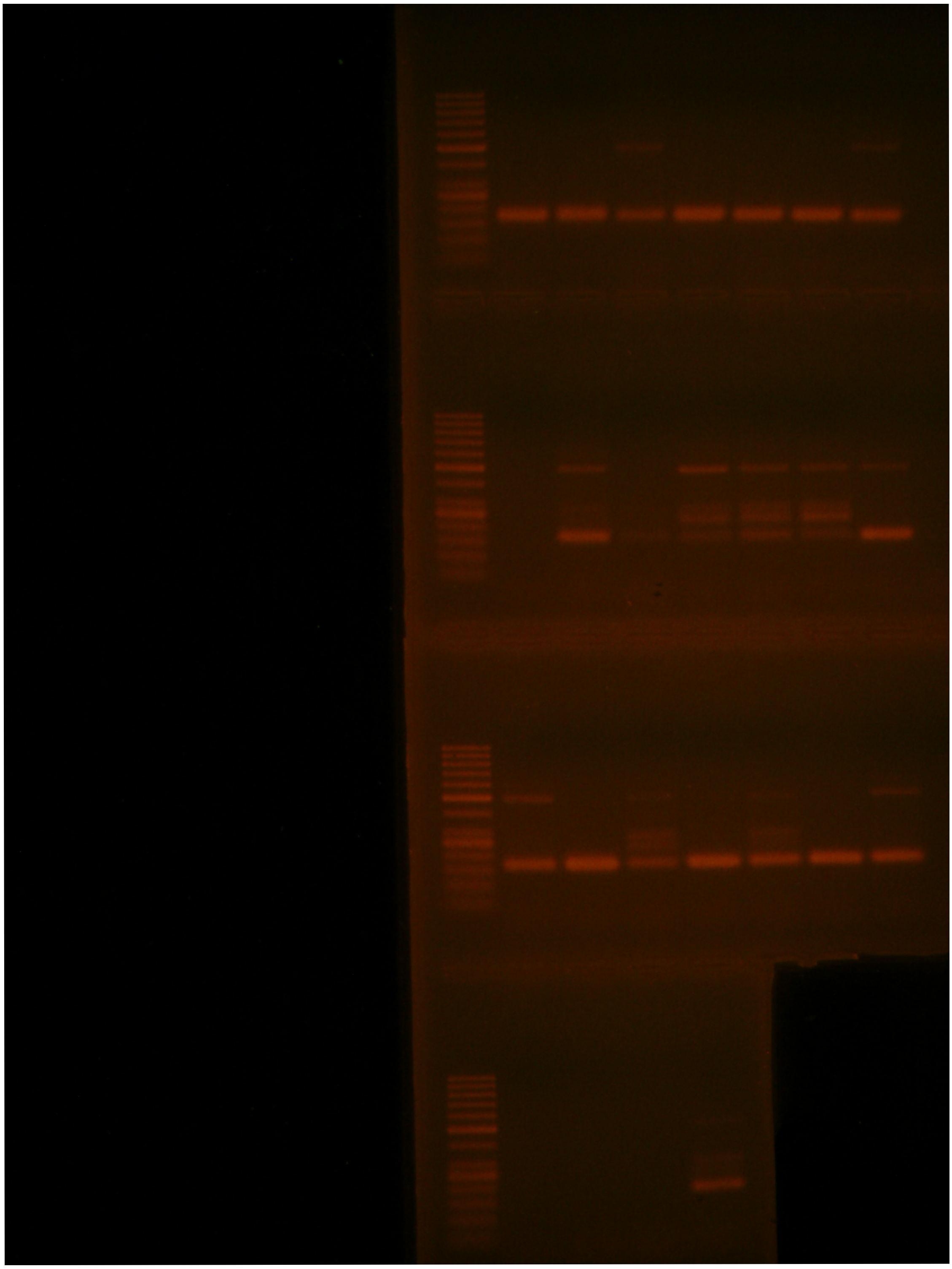

1 2 3 4 5 6 7 8 9

1 - 9 Unrelated samples

1 - 9 Unrelated samples

RPL22B intron

Rpl22

M

Mutclamp2

+ -

4 - 9 Unrelated samples

1 - 9 Unrelated samples

Originals to Fig 4A part 2- SPT 15 and RPL22B detection

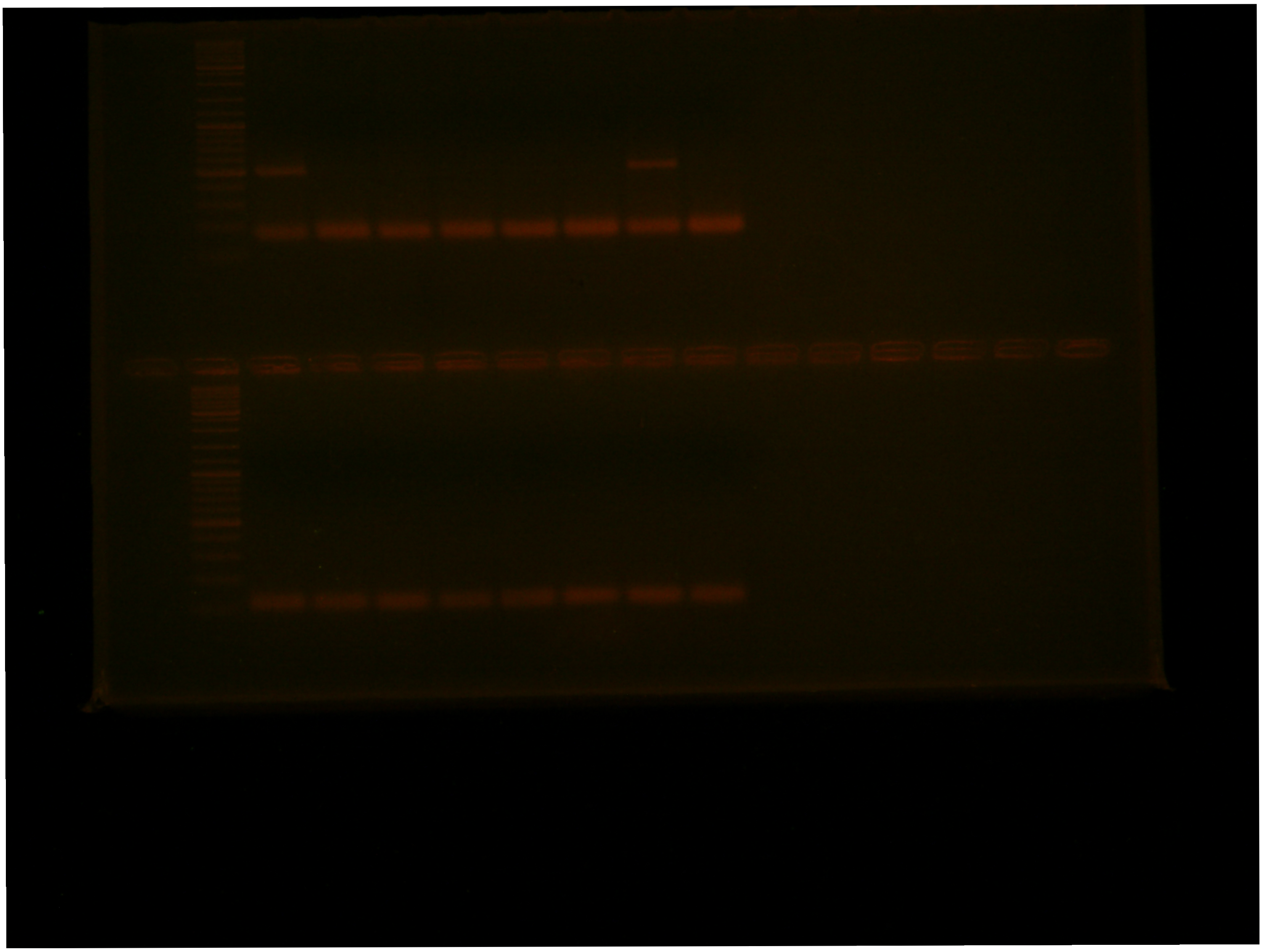

1 2 3 4 5 6 7 8 9 10 11 12 13 14 15 16

RPL22B intron			3-4 BPmut3		5 - 6 BPmut4			9-10 WT		
Rpl22	1 Empty	2 M	+	-	+	-	7- 8 unrelated samples	+	-	11-16 Empty

Upper part - RPL22B
Lower part - SPT15

Originals to Fig 4A part3- SPT 15 and RPL22B detection

A

B

C

D

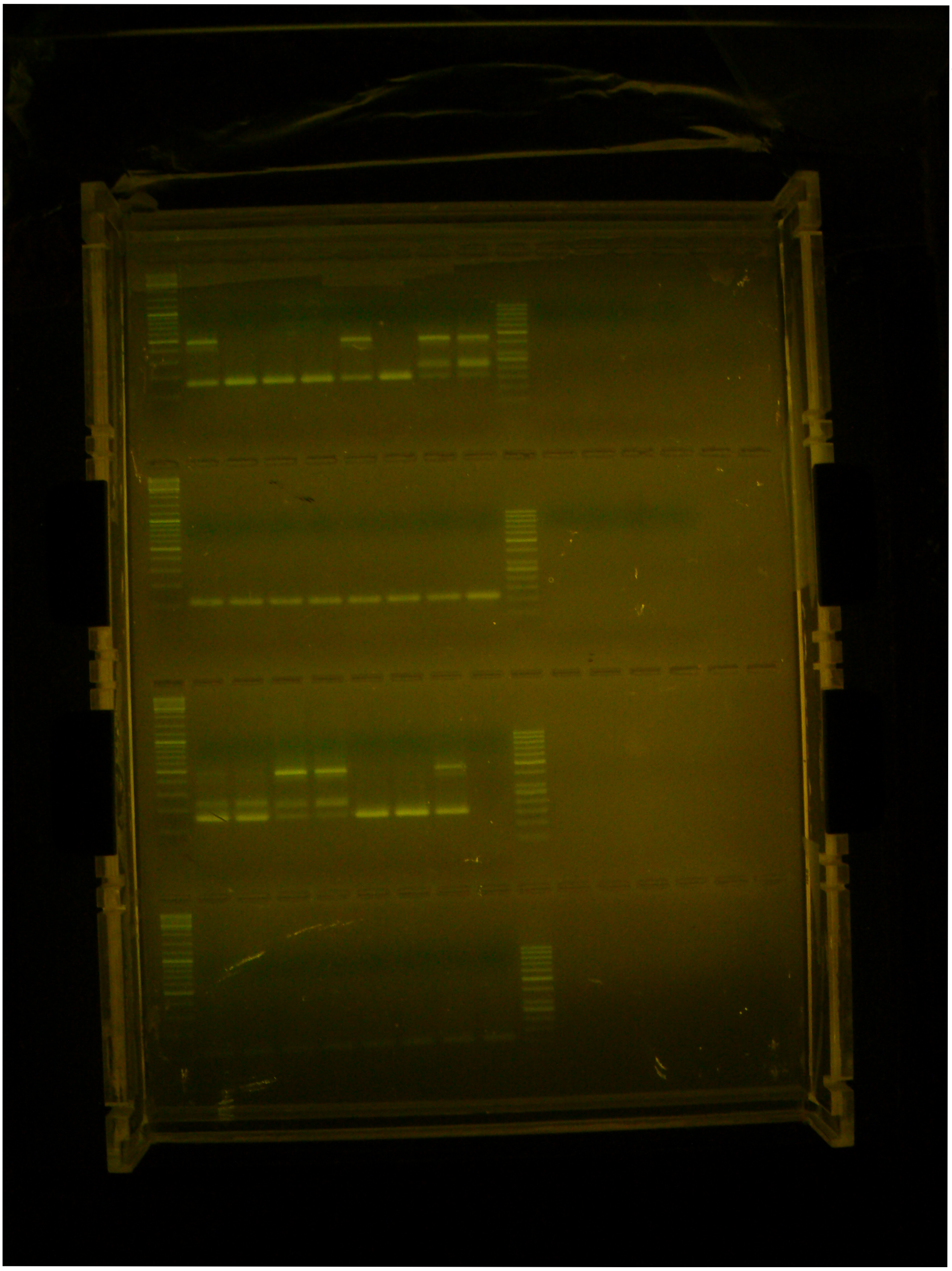

1 2 3 4 5 6 7 8 9 10 11 12 13 14 15 16

RPL22B intron	M	WT		3'CP BPmut4		dLoop2		3'CP dLoop2		M	11-14 neg. controls	15-16 Empty
Rpl22		+	-	+	-	+	-	+	-			

A - RPL22B
B- SPT15
C and D - unrelated samples

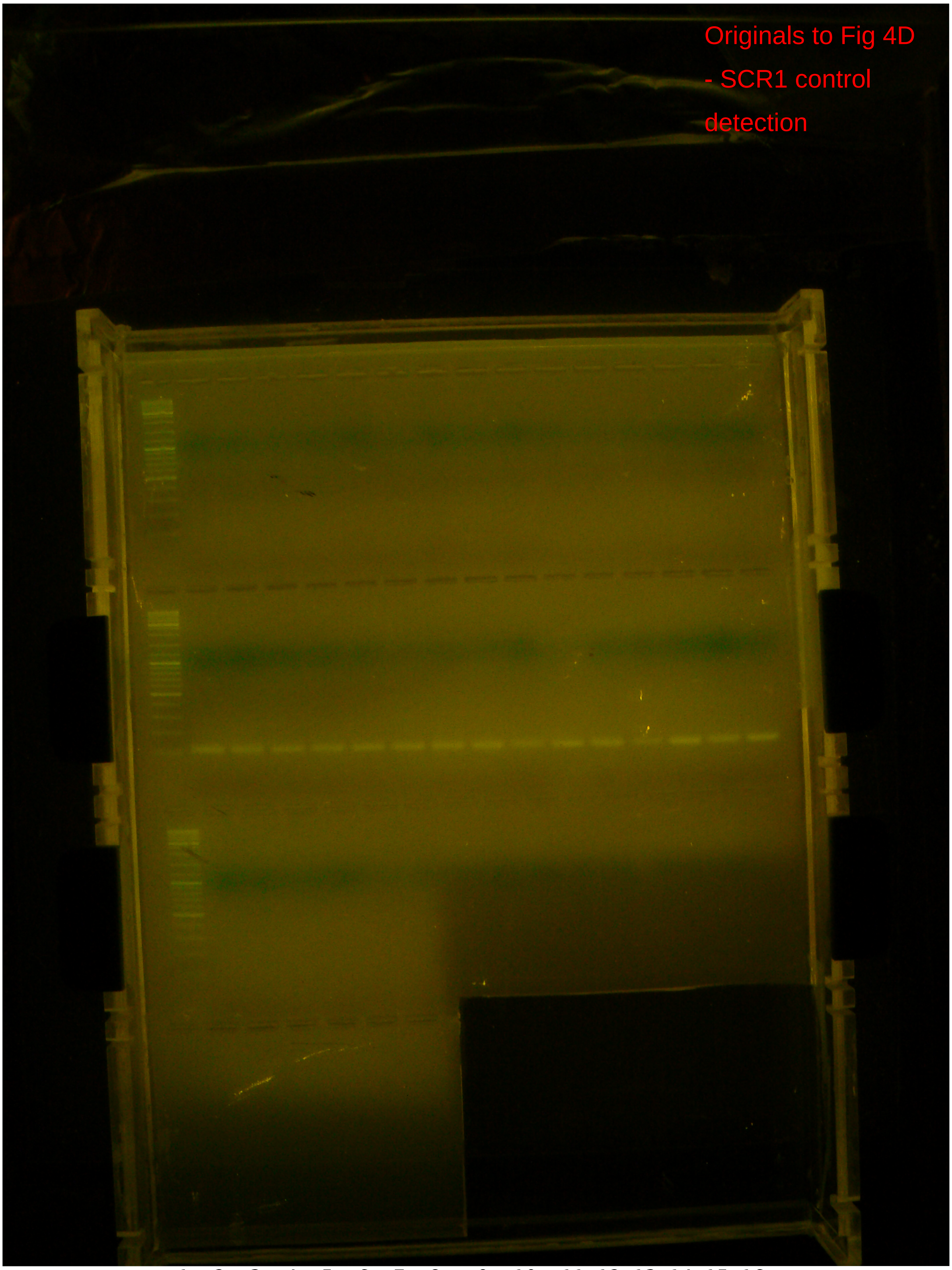

A

B

C

D

1 2 3 4 5 6 7 8 9 10 11 12 13 14 15 16

B-SCR1 detection

RPS17B intron		WT		transplant		unrelated samples										
Rpl22	M	+	-	+	-											
	1	2	3	4	5	6	7	8	9	10	11	12	13	14	15	16

A, C and D - unrelated samples

Originals to Fig 4D - transplant intron detection

A

B

C

D

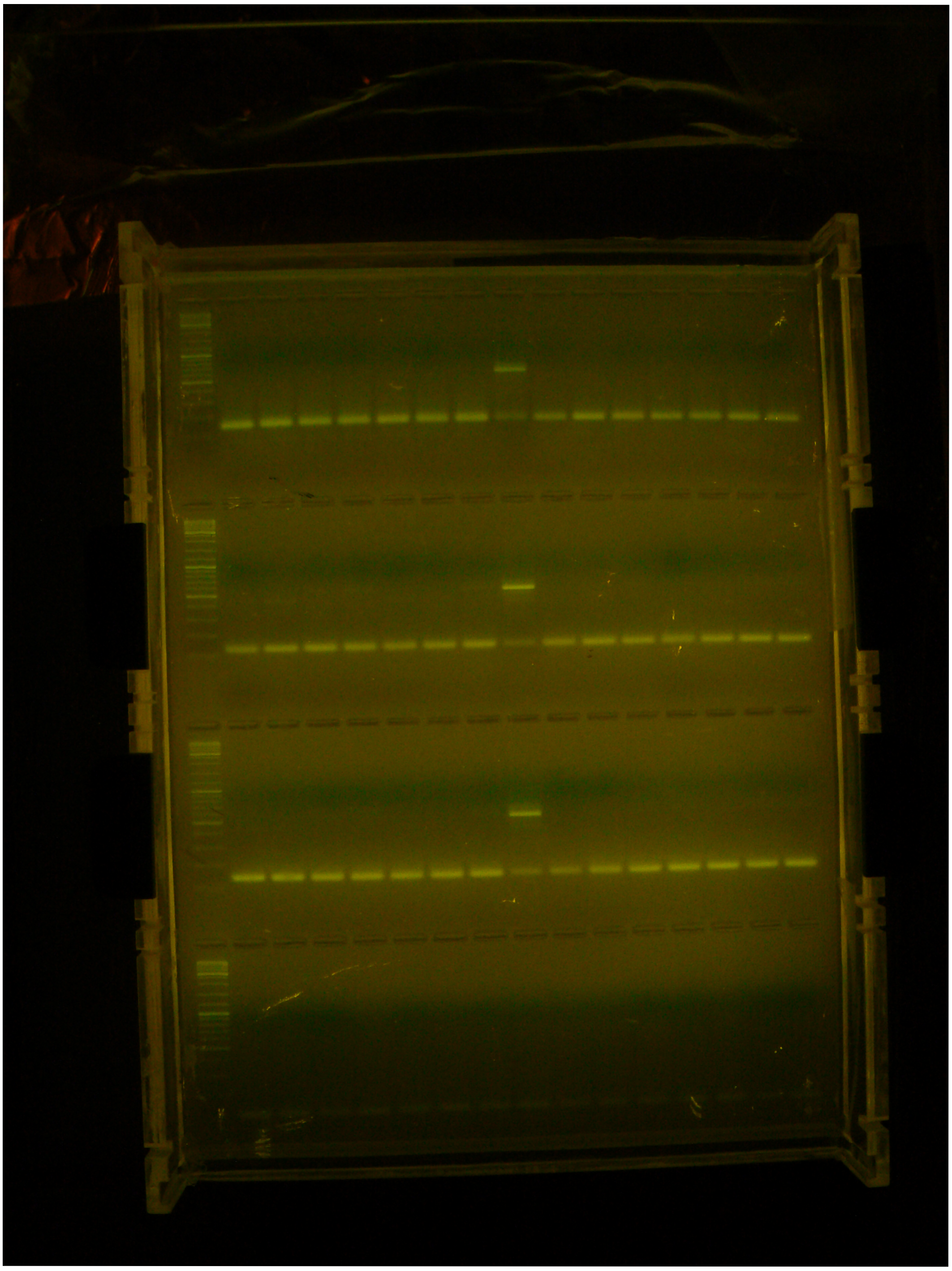

1 2 3 4 5 6 7 8 9 10 11 12 13 14 15 16

			WT		transplant		
A	*RPS17B* intron Rpl22	M	+	-	+	-	unrelated samples
		1	2	3	4	5	6 7 8 9 10 11 12 13 14 15 16

B, C and D - unrelated samples

Originals to Fig 5A - control SPT15 detection

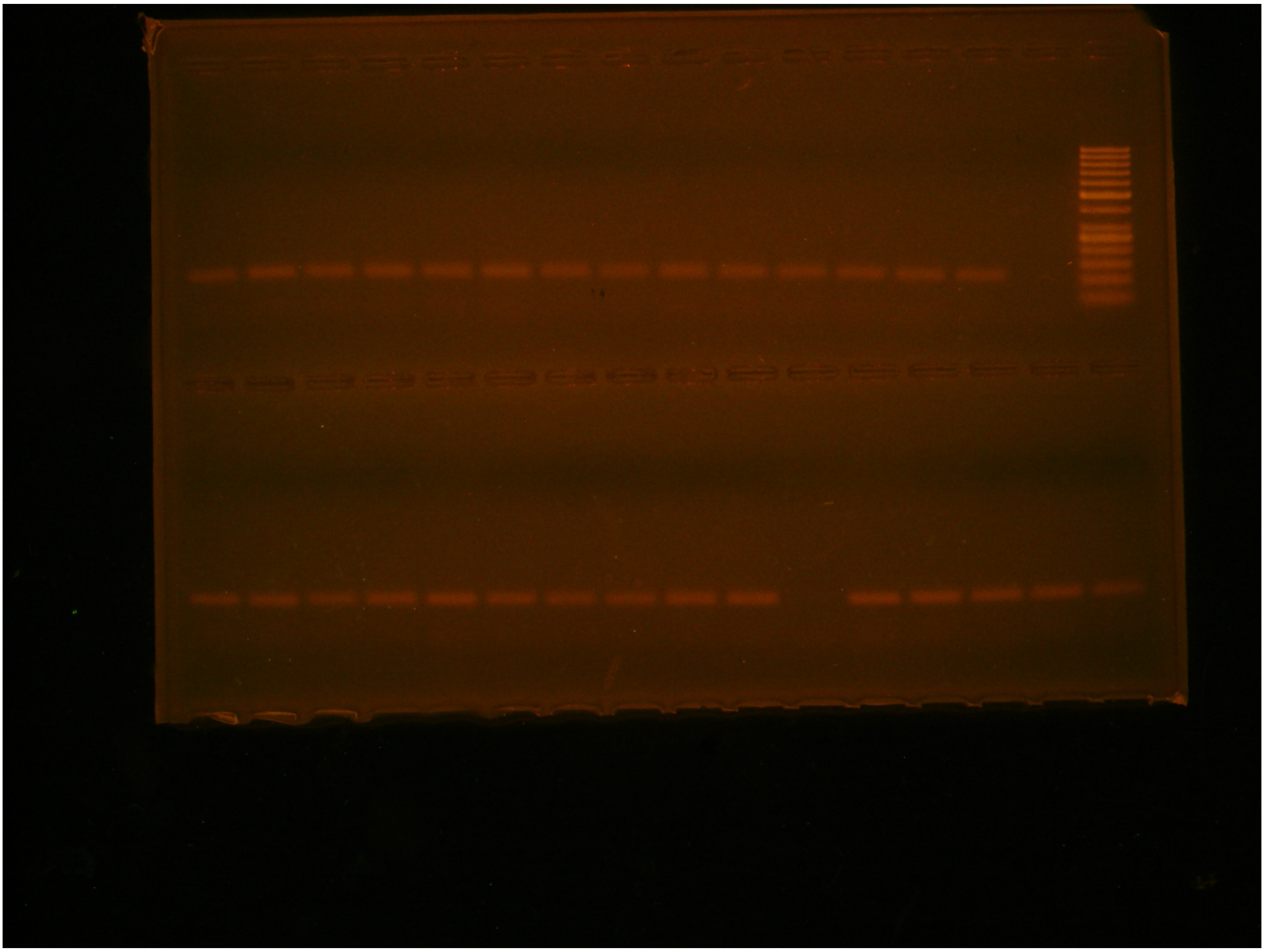

RPL22B intron	Δ Alt 5'ss		Alt 5'ss cons		5'ss cons		WT		Both 5'ss cons		5'CP Alt 5'ss cons		WT		neg ctrl	M
Rpl22	+	-	+	-	+	-	+	-	+	-	+	-	+	-		

Unrelated samples

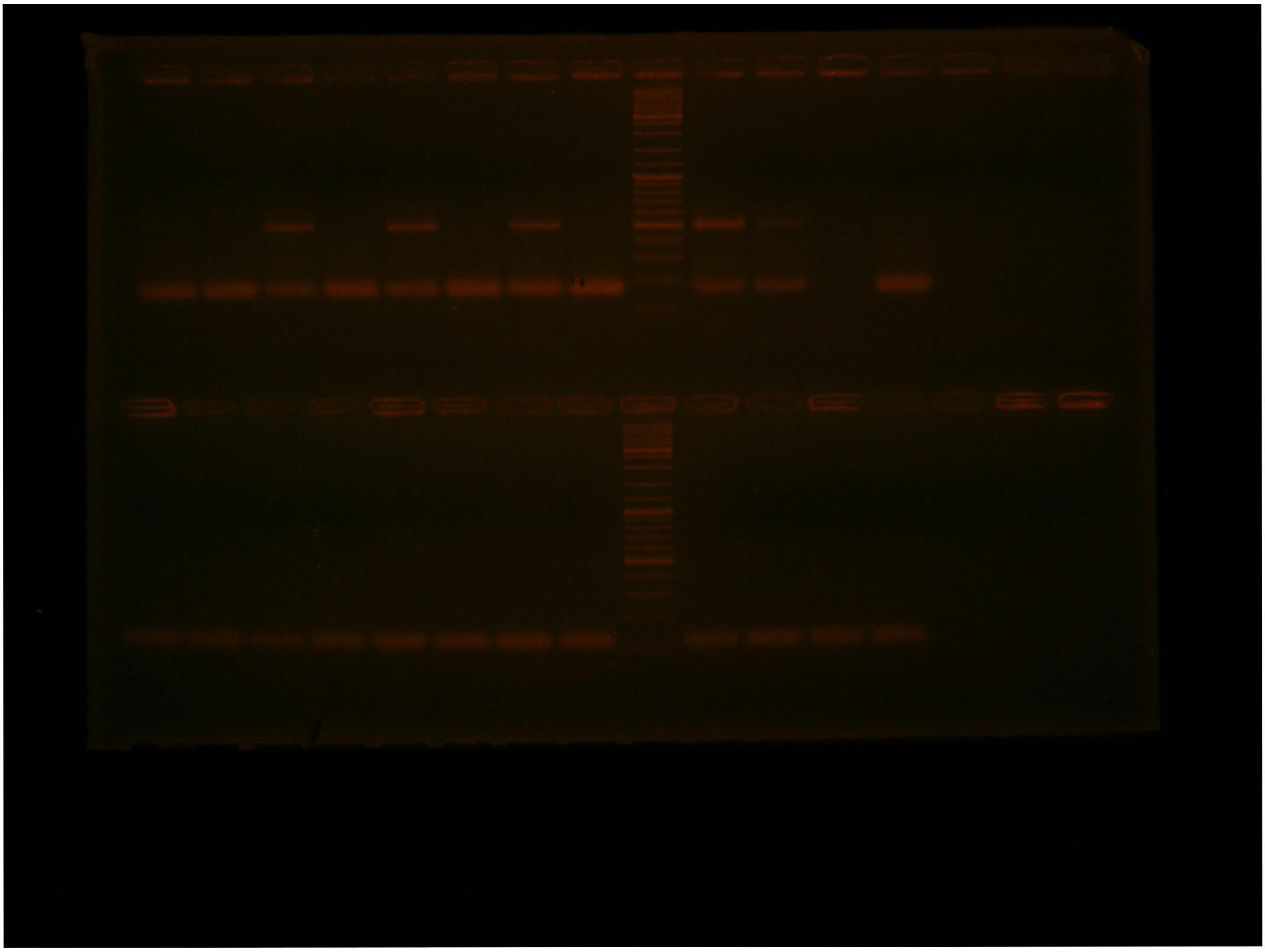

1 2 3 4 5 6 7 8 9 10 11 12 13 14 15 16

RPL22B intron	Clamp 'Loose'		Clamp 'Scramble'		Clamp 'Flip'		WT		M	10-13 unrelated samples	NRT	NTC	water
Rpl22	+	-	+	-	+	-	+	-					

Upper part - RPL22B
Lower part - SCR1

Originals to Fig 5Cpart2 - RPL22B and SCR1 detection

Originals to Fig 5D - RPL22B and SCR1 detection

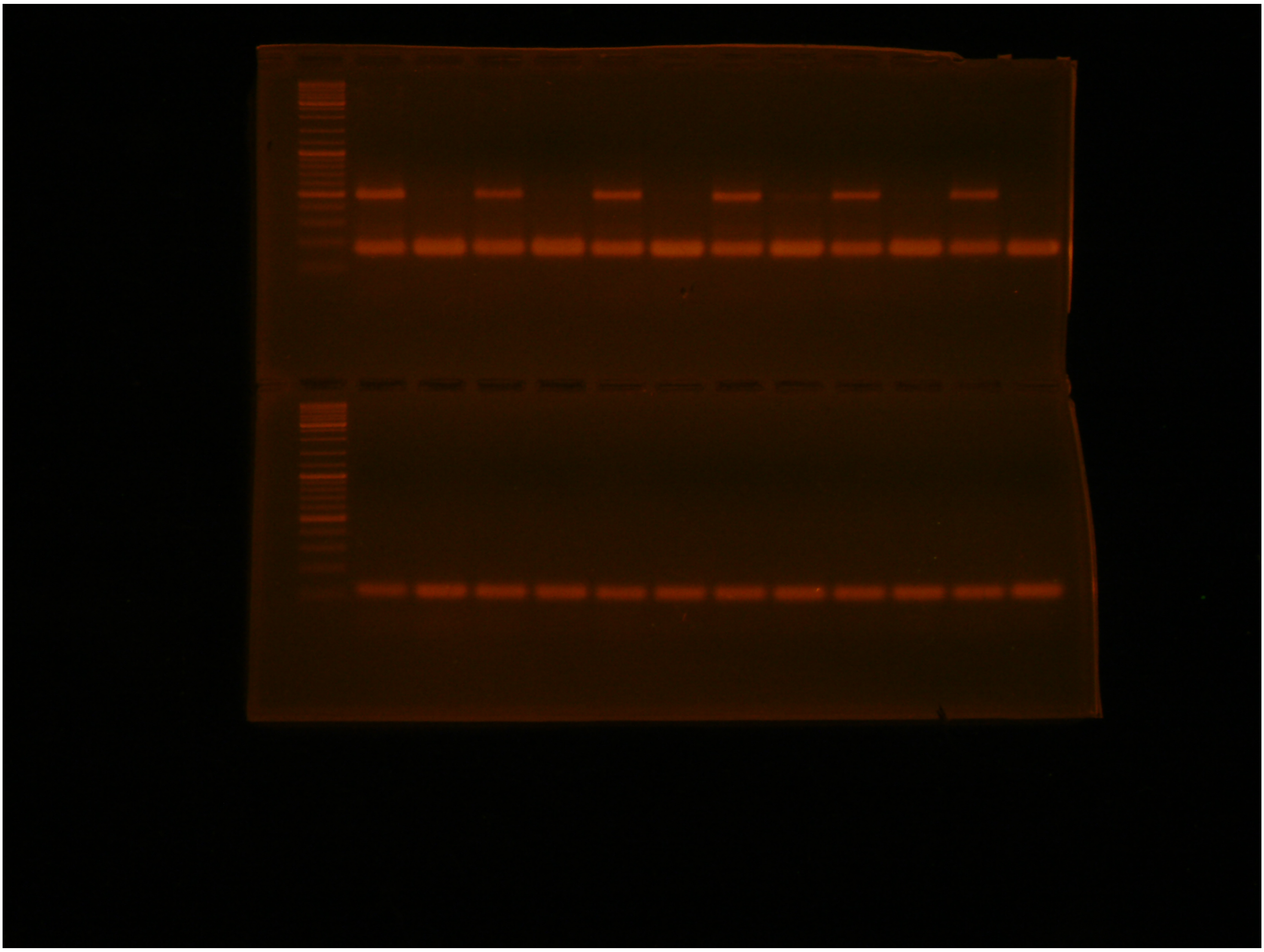

RPL22B intron		WT		US RIL AAAC		DS RIL CAUU		ΔRIL		ΔDS RIL		ΔUS RIL	
Rpl22	M	+	-	+	-	+	-	+	-	+	-	+	-

Upper part - RPL22B
Lower part - SCR1

Originals to Fig 7D - Splicing analysis

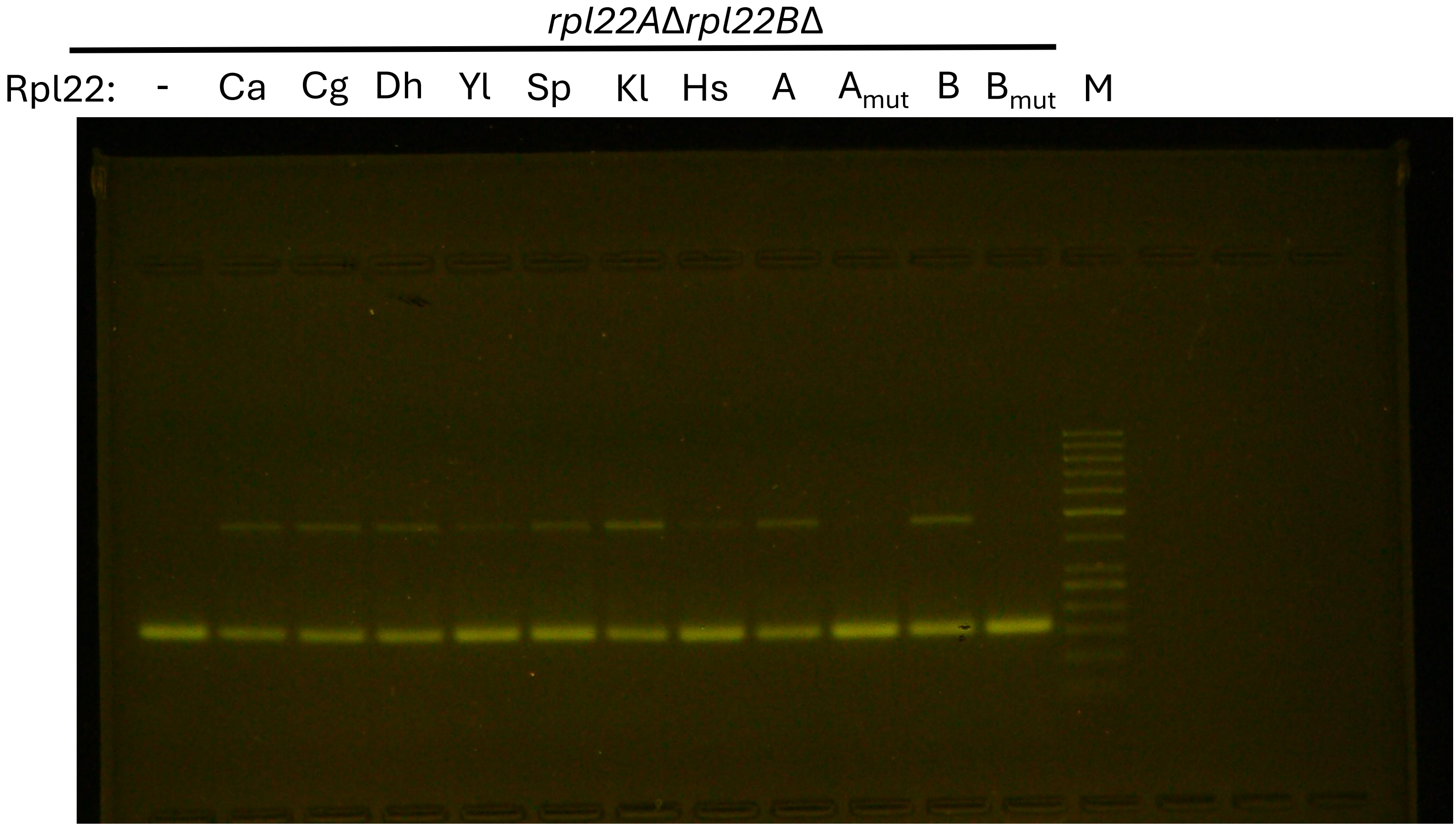

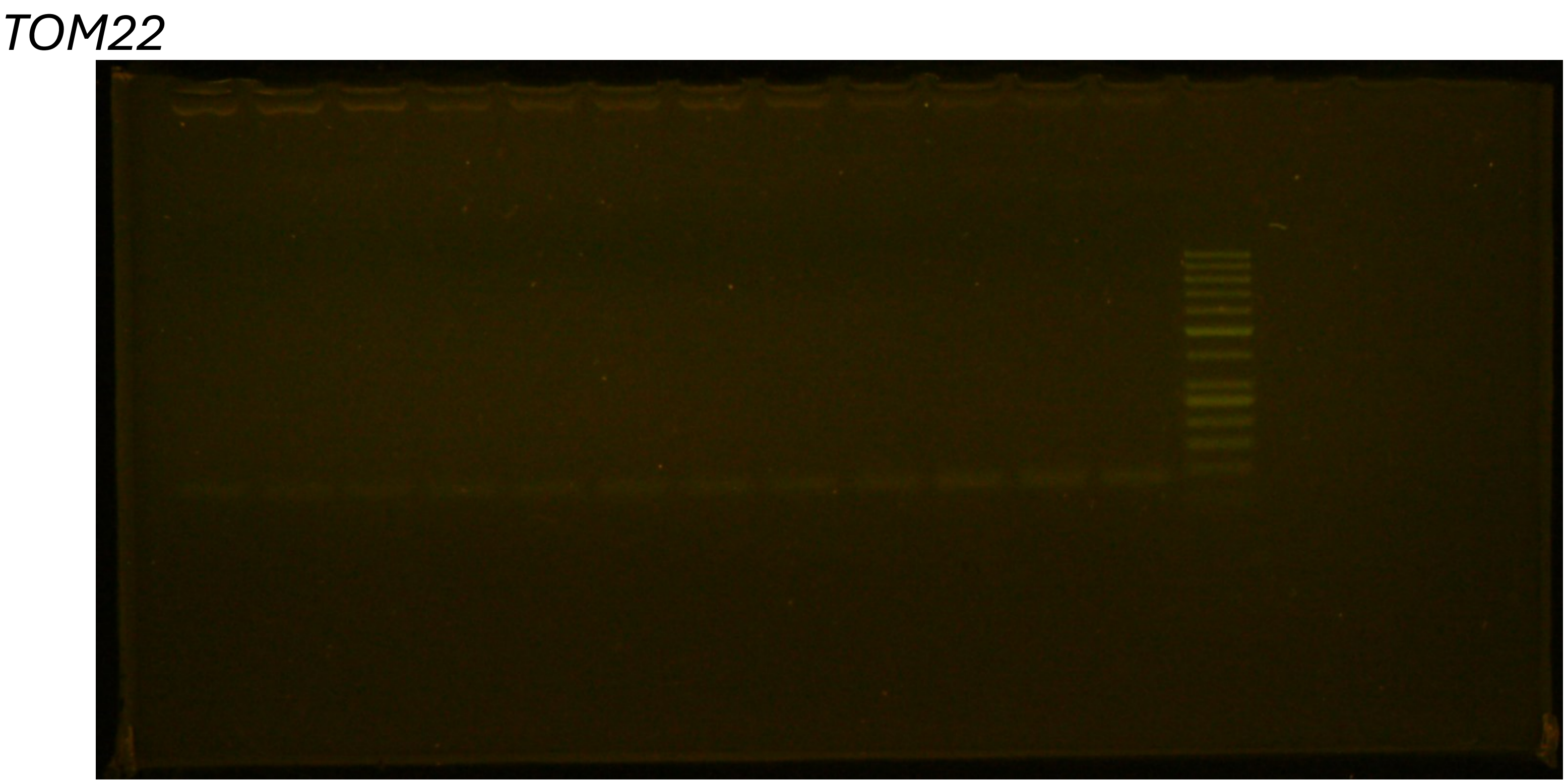

Originals to Fig S10 - Protein production

A

WT

Rpl22: Ca Cg Dh Yl Sp Kl Hs A A_{mut}

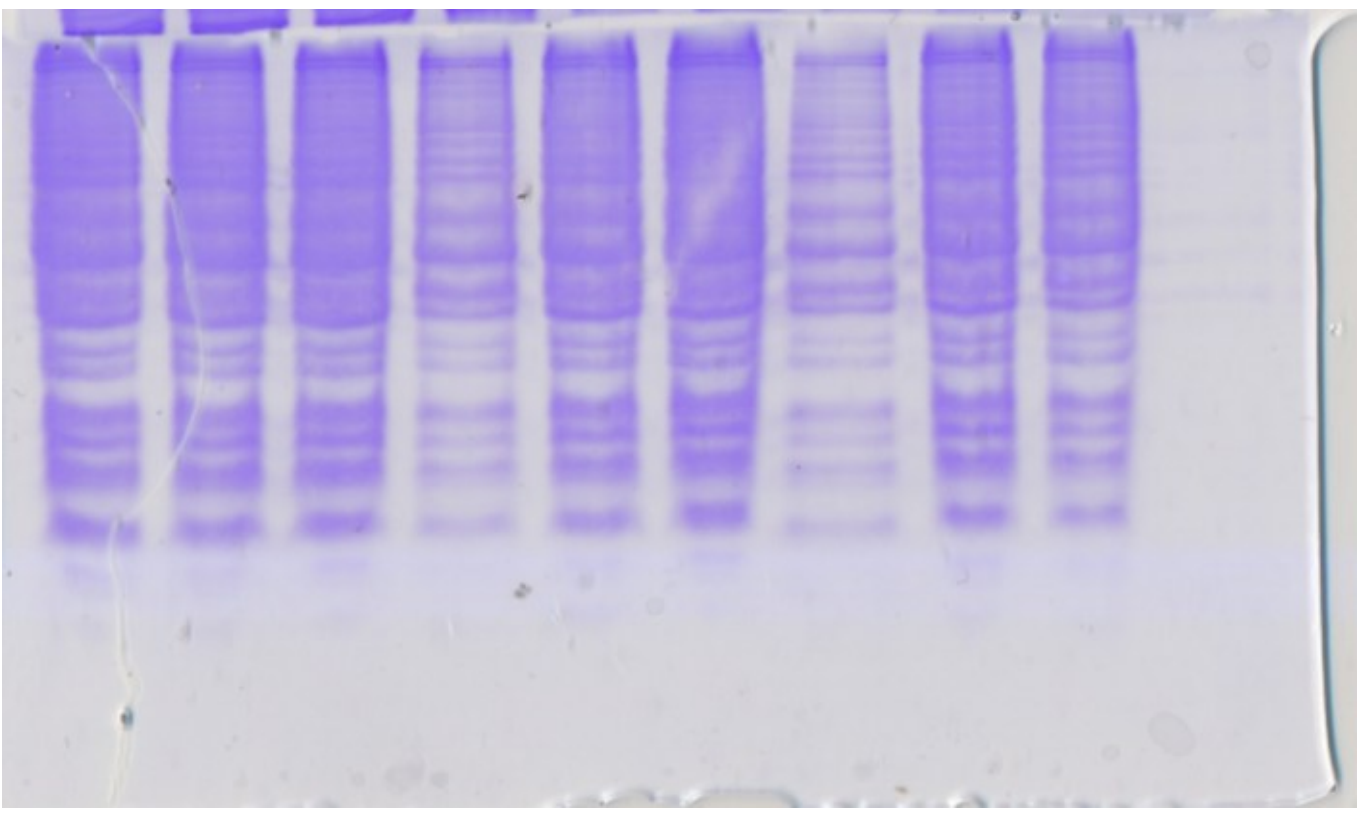

WT | *rpl22AΔrpl22BΔ*

B B_{mut} - Kl Hs B B_{mut} A_{mut}

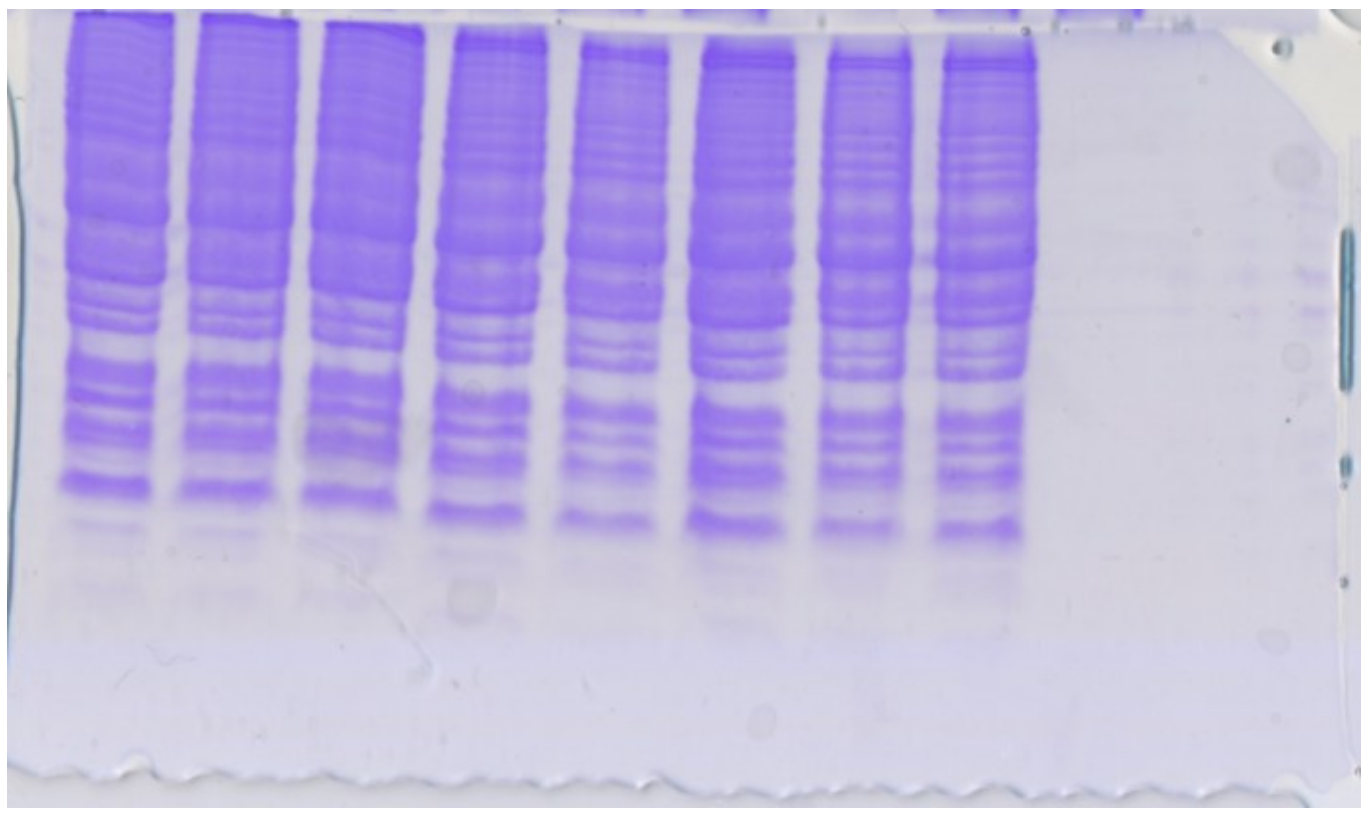

rpl22AΔrpl22BΔ

Rpl22: - Ca Cg Dh Yl Sp X A

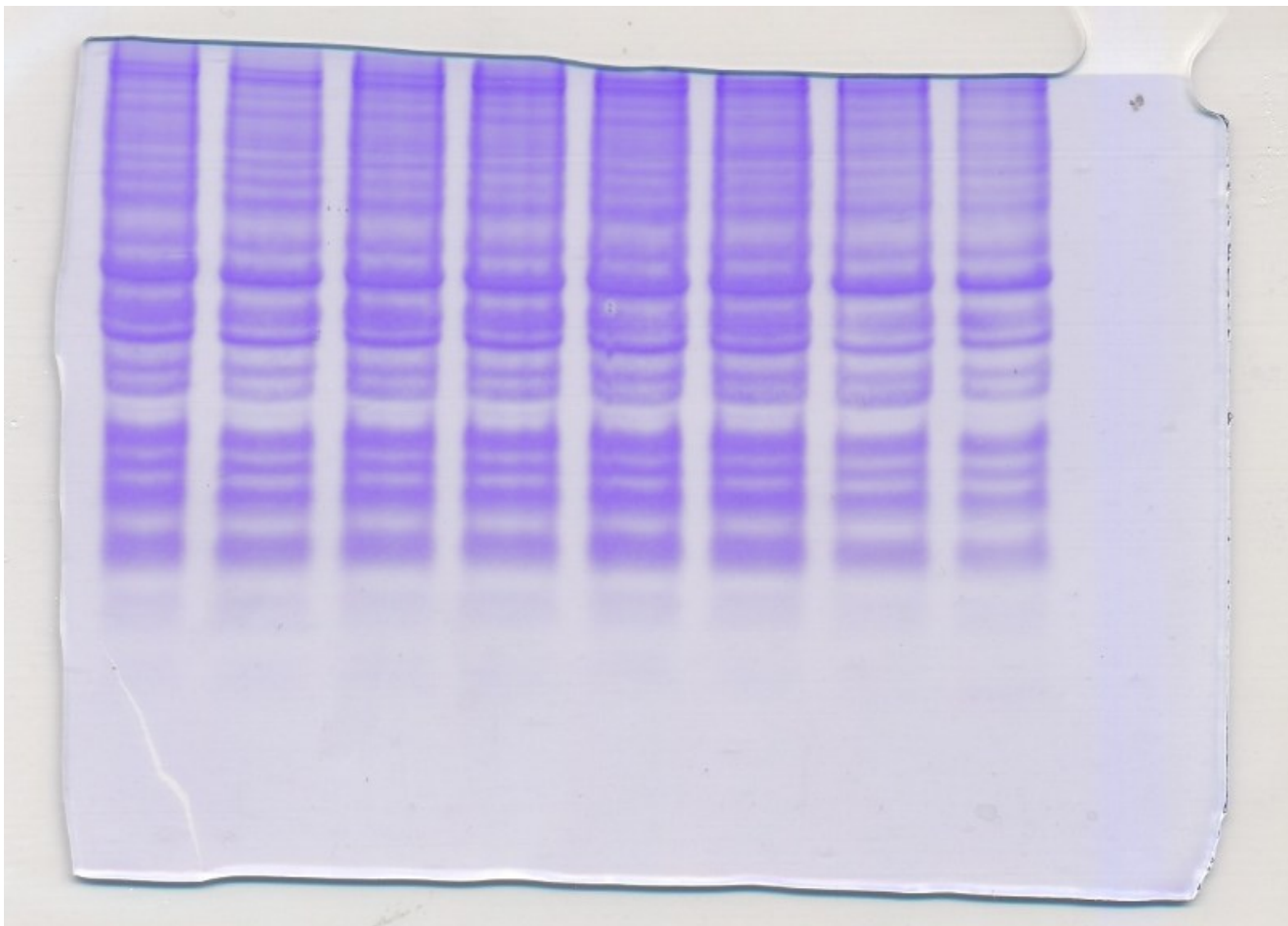

B

rpl22AΔ

Rpl22: Ca Cg Dh Yl Sp Kl Hs A A_{mut}

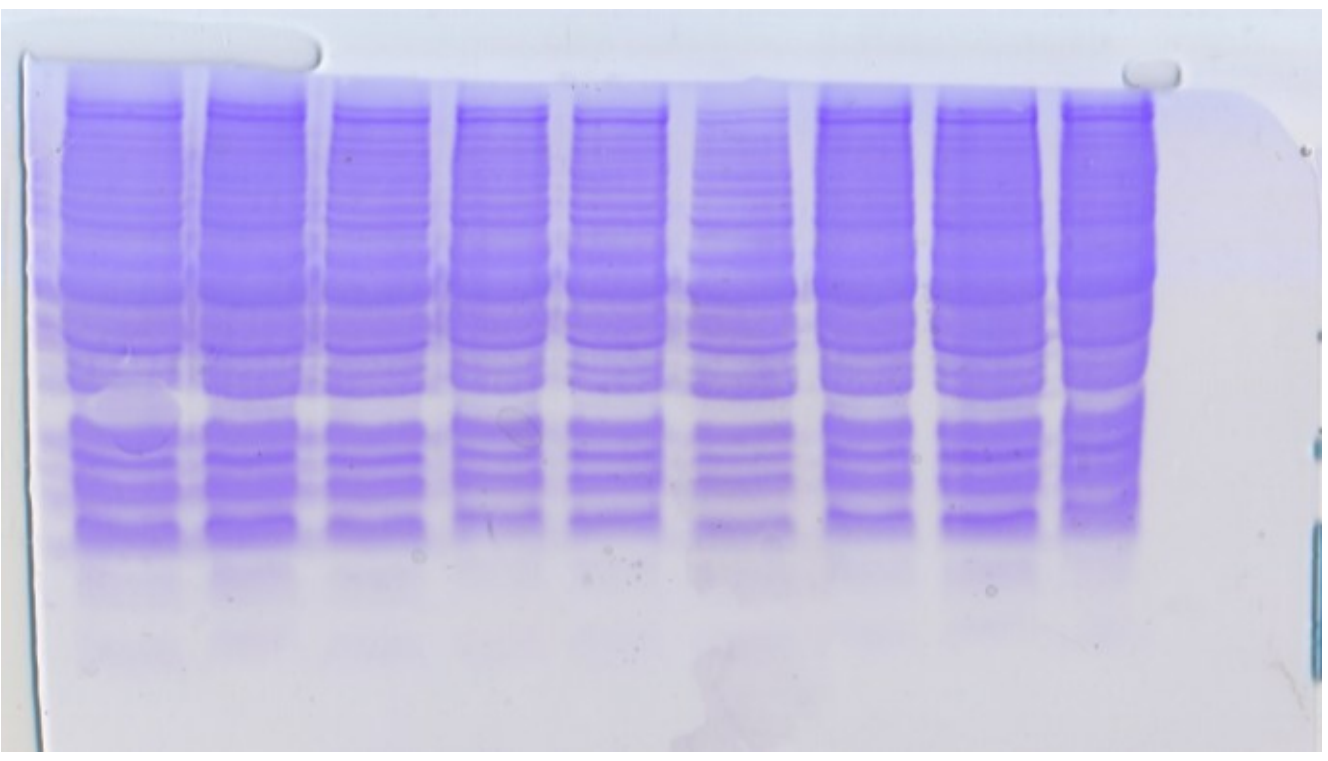

B **C**

rpl22AΔ | *rpl22AΔrpl22BΔ*

B B_{mut} - A_{mut} B B_{mut}

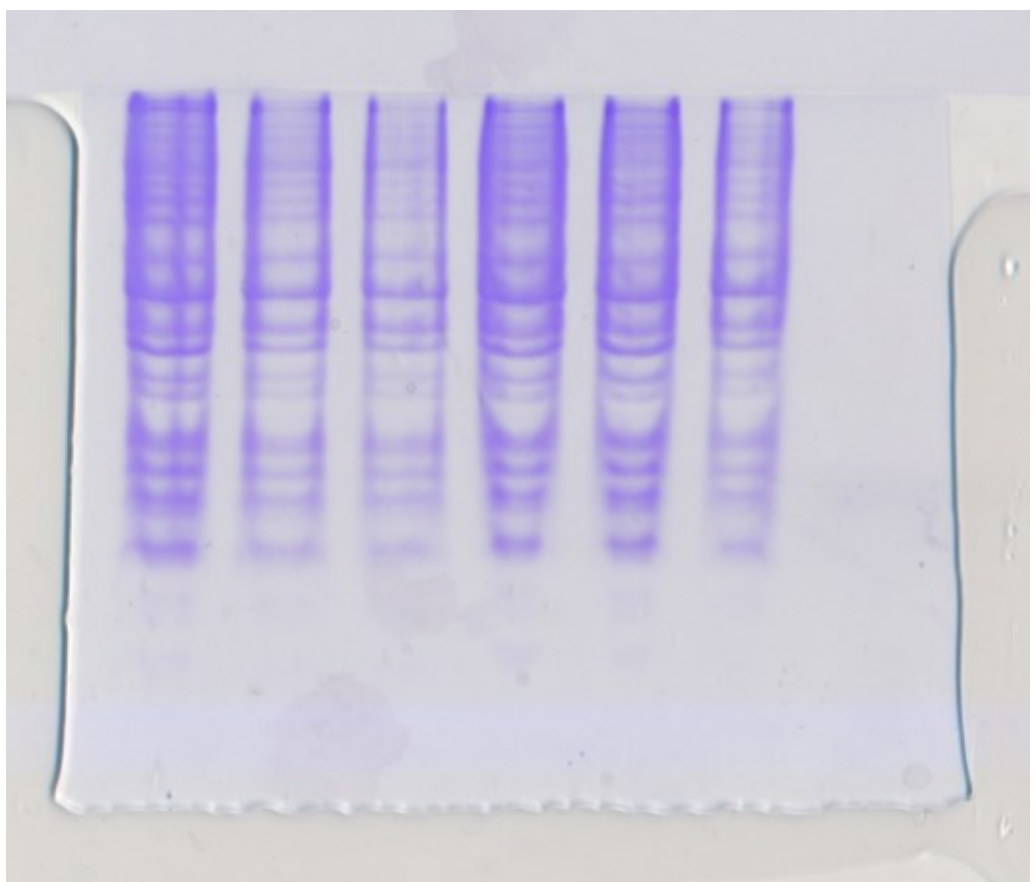

C

rpl22AΔrpl22BΔ

Rpl22: - Ca Cg Dh Yl A Hs Kl Sp

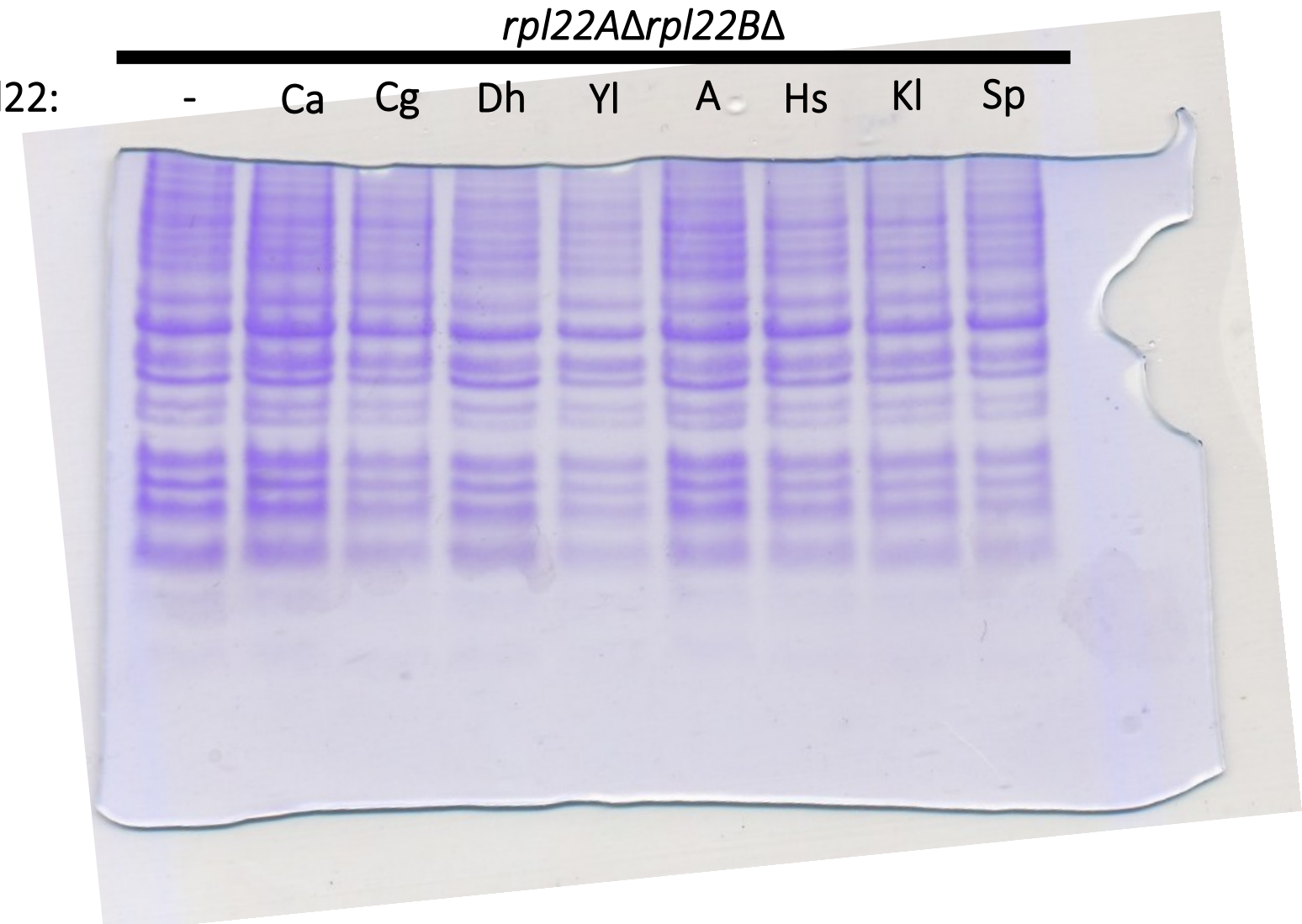

Originals to Fig S10 - Westerns

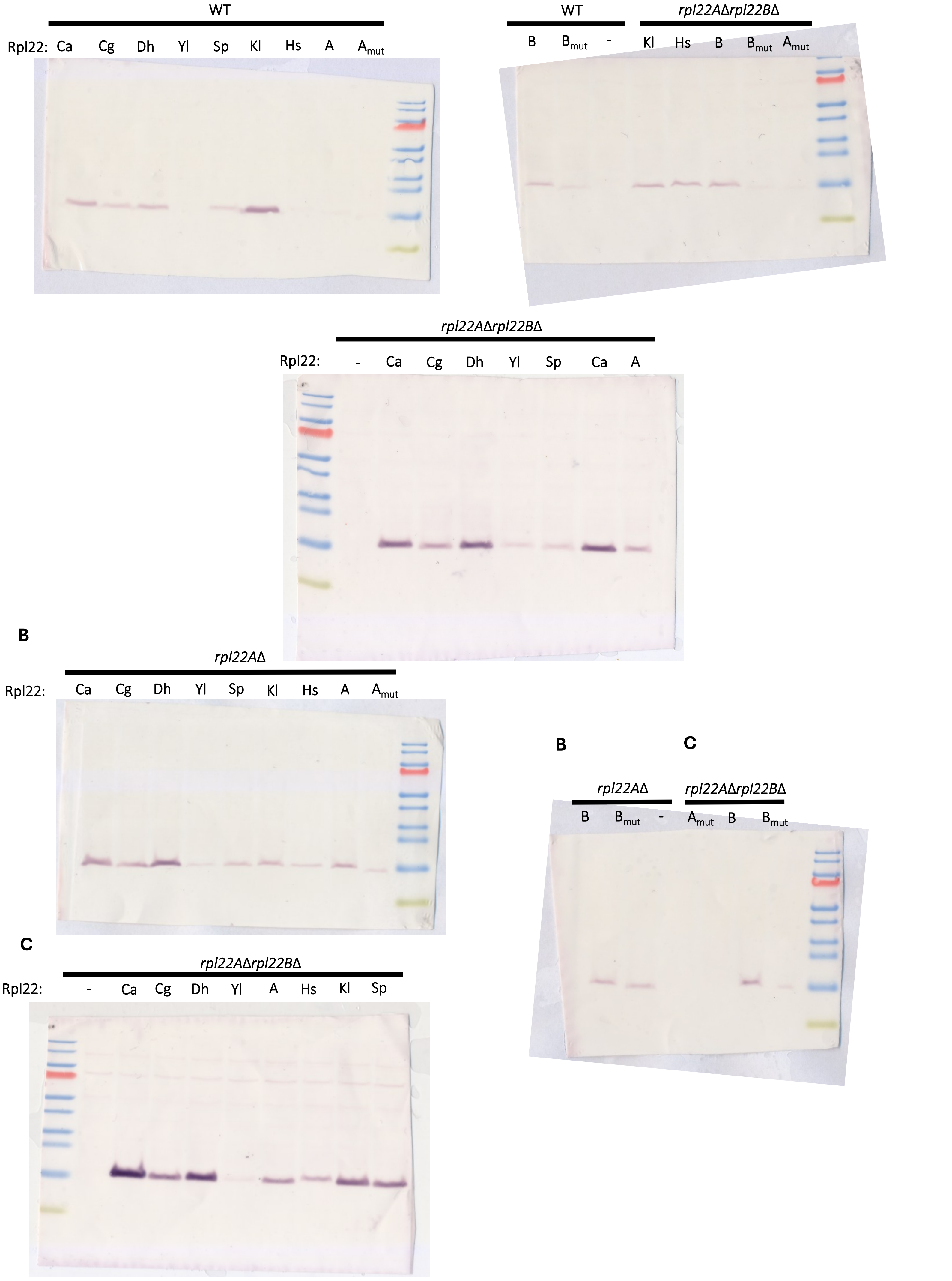

Supplement: Supplementary file 10 [file pone.0359197.s011.pdf]
