# Supplementary material for: Intronic RNA of yeast RPL22 paralogs acts as an allosteric switch
Source: PLoS One. 2026 Oct 1;21(10):e0359197. doi: 10.1371/journal.pone.0359197 (PMC13630204; doi:10.1371/journal.pone.0359197)
Supplement: Supplementary file 11 [file pone.0359197.s012.pdf]

MFE secondary structure

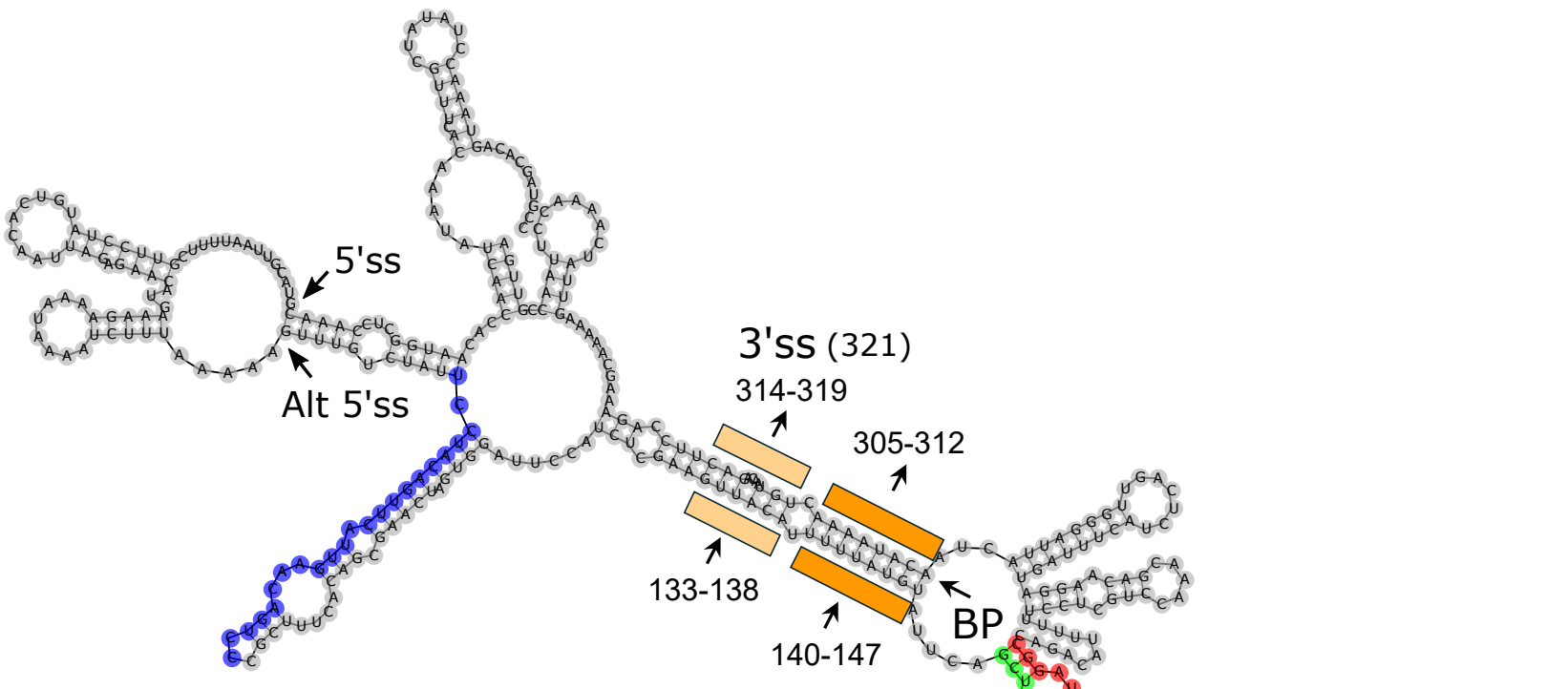
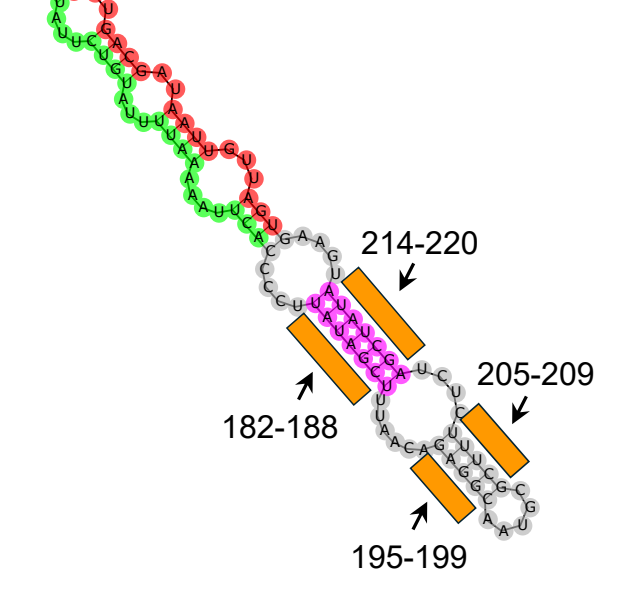

BP probabilities

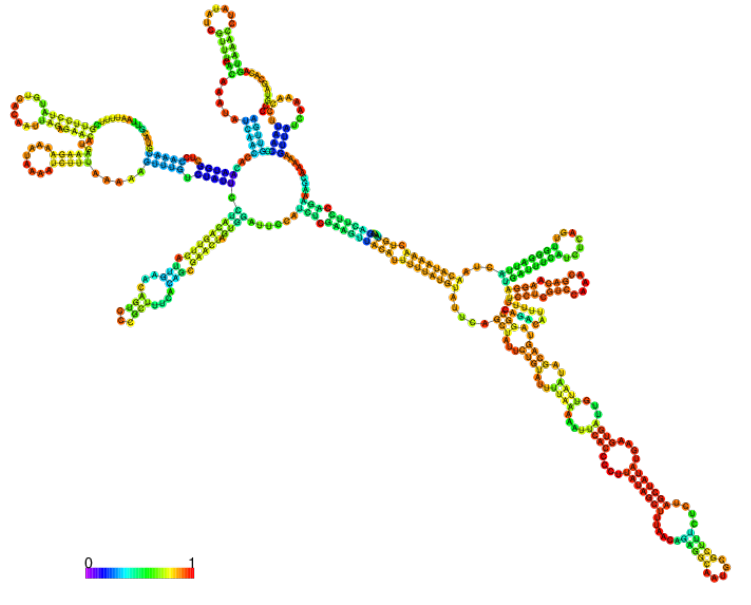

S12 Fig Secondary structure prediction of the *RPL22B* intron by RNAfold is supported by DMS reactivity data published previously.

The prediction of inhibitory structure for the WT *RPL22B* intron is the same as shown in Fig 1B (Inhibitory structure; upper left). The key sequence elements described in Fig 1A are labeled red (conserved region, green (3'complementary element), blue (5'complementary element) and violet (Clamp). The nucleotides that were found by Rangan et al. in helical segments (> 70% confidence estimate stems; [7]) using DMS reactivity profiling *in vivo* are indicated by boxes located near the structure. The DMS data match exactly two helical segments (ochre) in the "main stem loop" and one neighboring the branch point. Nucleotides 133-138/ 314-319 (light ochre) involve a bulge in the prediction, which is in the vicinity of the 3'ss. The secondary structure diagram colored according to raising base pair probabilities (blue to red) as predicted by RNAfold is shown in the inset.

[7] Rangan R, Huang R, Hunter O, Pham P, Ares M Jr, Das R. Comprehensive analysis of Saccharomyces cerevisiae intron structures in vivo. Nat Struct Mol Biol. 2025;32:1488-1502. https://doi: 10.1038/s41594-025-01565-x.
